# MitoQ Has Diverse Effects on H_2_O_2_-Induced Oxidative Stress and the NRF2 Signalling Pathway in Aortic Smooth Muscle Cells of Different Origins

**DOI:** 10.3390/cells15161499

**Published:** 2026-08-20

**Authors:** Simon Cornelius Haas, Bingchen Hou, Andreas Sebastian Peters, Johannes Hatzl, Dittmar Böckler, Susanne Dihlmann

**Affiliations:** 1 Klinik für Vaskuläre und Endovaskuläre Chirurgie, Universitätsklinikum Heidelberg, Im Neuenheimer Feld 420, 69120 Heidelberg, Germany; simon.c.haas@gmail.com (S.C.H.); andreas.peters1@med.uni-heidelberg.de (A.S.P.); johannes.hatzl@med.uni-heidelberg.de (J.H.); dittmar.boeckler@med.uni-heidelberg.de (D.B.); 2 Vaskuläre Biomaterialbank Heidelberg (VBBH), Im Neuenheimer Feld 420, 69120 Heidelberg, Germany

**Keywords:** oxidative stress, NRF2 signalling, vascular smooth muscle cells, MitoQ, mitoquinone mesylate, abdominal aortic aneurysm

## Abstract

**Highlights:**

This in vitro study presents evidence that long-term/low-dose treatment with MitoQ, a mitochondria-targeted derivative of coenzyme Q10, improves the antioxidative stress response and reduces ROS production, particularly in VSMC derived from abdominal aortic aneurysm (AAA), without affecting healthy VSMC. In line with previous findings, the mechanism of action involves activation of NRF2 signalling, which was found to be affected in AAA.

**What are the main findings?**

- NRF2 signalling is affected in AAA tissues.
- VSMC derived from AAA are less vulnerable toward high MitoQ doses than healthy VSMC.
- Long-term/low-dose MitoQ treatment reduces ROS production, particularly in VSMC derived from AAA.

**What are the implications of the main findings?**

- Long-term/low-dose MitoQ treatment has a cytoprotective effect on VSMC derived from AAA, without affecting healthy cells.
- Given previous clinical studies demonstrating that treatment with MitoQ improves vascular function, supplementation could also be considered for patients with a small AAA to reduce oxidative stress-induced damage to VSMCs.

**Abstract:**

Oxidative stress plays a central role in the development and progression of abdominal aortic aneurysms (AAA), as it severely impairs the function and survival of vascular smooth muscle cells (VSMCs). MitoQ (mitoquinone mesylate), a mitochondria-specific antioxidant, was shown to reverse age-related arterial stiffening and improve vascular endothelial function, among other things, by interacting with the NRF2 signalling pathway. The aim of this study was to compare how long-term treatment with low doses of MitoQ affects the NRF2 stress response in VSMCs, derived from different origins (AAA-SMC, healthy aortic SMC, and immortalized VSMC (iHAoSMC)). We found a significant reduction in NRF2 and KEAP1 levels in the aortic wall of patients with AAA, accompanied by increased 8-OHdG levels, indicating defects in the response to oxidative stress. In contrast, relative NRF2 expression in tissue extracts and VSMC-enriched areas was higher in patients with AAA than in healthy aortic tissue. In vitro, baseline NRF2 protein levels were significantly higher in AAA-SMC and in iHAoSMC than in VSMC from healthy aorta, whereas NRF2 activity did not differ between AAA-derived and healthy VSMC. AAA-derived SMC were found to be less vulnerable against toxic concentrations of MitoQ than healthy VSMC, and the cell viability was differentially affected by H_2_O_2_. Acute oxidative stress by H_2_O_2_ increased NRF2 activity in AAA-SMC and iHAoSMC, but not in healthy VSMC. Pre-treatment of the cells for 7 days with low-dose (10 nM) MitoQ resulted in significantly increased NRF2 activity in AAA-SMC and iHAoSMC, but not in healthy VSMC, which was accompanied by a significant reduction of ROS production, particularly in AAA-derived SMC. Our data demonstrate that prolonged treatment with low doses of MitoQ has a protective effect, particularly on VSMCs from AAA, without affecting healthy aortic VSMCs. Moreover, immortalized cells can be used as a model for investigating oxidative stress responses in AAA-SMC, even though they do not react in exactly the same way. Overall, our findings confirm the cytoprotective potential of MitoQ to limit oxidative stress, particularly in AAA-SMC that is clinically observed in the abdominal aneurysm wall.

## 1. Introduction

Aortic smooth muscle cells (SMCs) in the aortic media play a central, pathological role in abdominal aortic aneurysm (AAA) by undergoing phenotypic transition from a contractile to a synthetic state. Single-cell RNA sequencing has revealed that synthetic VSMC are highly heterogeneous and can transdifferentiate into multiple pathogenic phenotypes. This phenotypic diversity contributes to apoptosis, inflammation, and excessive extracellular matrix (ECM) degradation [1]. Oxidative stress, primarily from NADPH oxidases (NOX) and reactive oxygen species (ROS), promotes these changes, leading to elastin degradation and structural collapse. The progressive loss of functional aortic SMCs weakens the aortic wall, further driving dilatation and potential rupture [2]. The phenotypic switch of SMCs has been extensively studied in atherosclerosis [3,4] and is considered to be a driver of aneurysm growth, although the sequential pathophysiology of AAA is unclear [2,5,6].

Oxidative stress is considered a physiological condition defined by an imbalance between the excessive production of unstable, oxygen-containing molecules (ROS or free radicals) and the body’s antioxidant defense mechanisms’ ability to neutralize them. The primary factors for induction of oxidative stress range from obesity, poor diet, smoking, drinking alcohol, and taking certain medicines to exposure to environmental factors such as radiation, toxins, air pollution, pesticides, and sunlight [7]. ROS, including superoxide anion (O_2_^−^), hydrogen peroxide (H_2_O_2_), and hydroxyl radical (HO^.^), are natural byproducts of oxygen metabolism in the body and play an important role in cell signalling [8]. Within the aortic wall, increased ROS production, i.e., resulting from NOX, dysfunctional mitochondria, or inflammation, are considered major contributors to the phenotypic switching and loss of functional SMCs [8]. The effects of ROS on SMCs include modulation of cell cycle, apoptosis, DNA synthesis, expression of differentiation markers, and epigenetics, just to name a few [8].

Due to the wide range of effects that ROS exert on SMCs, the cells have a sophisticated system for regulating ROS levels and keeping homoeostasis. Within this system, the nuclear factor erythroid 2-related factor 2 (NRF2, encoded by NFE2L2) is considered the master regulator of the body’s antioxidant defenses, acting as a crucial transcription factor that activates genes for antioxidant and detoxification enzymes in response to oxidative stress, not only in SMCs but in virtually all cell types [9]. NRF2 abundance within the cell is tightly regulated, and its basal protein levels are usually low in unstressed conditions. This is achieved by a continuous sequestration by Kelch-like-ECH-associated protein 1 (KEAP1) and subsequent proteasomal degradation, ensuring that only the basal NRF2 target genes are maintained for housekeeping functions. Under stressed conditions, i.e., metabolic alterations, increased ROS production, autophagy disruption, or others, the cellular amount of NRF2 is temporarily or constitutively increased. ROS react with cysteines within KEAP1, thereby allowing NRF2 release and accumulation of newly synthesized NRF2, which migrates to the nucleus and activates the expression of cytoprotective genes [9]. Recently, single-cell RNA sequencing revealed that NRF2 regulates aortic SMC phenotypic switching in AAA. The authors further observed that the absence of NRF2 in aortic SMCs exacerbated AAA formation in an animal model. Moreover, downregulation of NRF2 promoted switching of the aortic SMCs, thereby enhancing the inflammatory response [10].

MitoQ (mitoquinone mesylate) is a mitochondria-targeted derivative of coenzyme Q10 (CoQ10) that was developed to accumulate in mitochondria more efficiently than CoQ10. It exerts its impact on cellular function mainly by reducing oxidative stress caused by overproduction of ROS within cells. Within the mitochondrial matrix, MitoQ is reduced and then reoxidized, thereby continually scavenging free radicals [11]. Being available as a dietary supplement, it shows promise for improving cardiovascular health by reducing oxidative stress and improving mitochondrial function [12]. MitoQ was shown to reduce age-related aortic stiffness and vascular fibrosis induced by environmental factors in mouse models [13,14]. However, whether MitoQ can improve the function of aortic SMCs, particularly SMCs derived from human AAA, is unknown so far. Interaction of MitoQ with the NRF2 pathway has been suggested to occur within animal SMCs, [15] endothelial cells (ECs), [16] and human cardiomyocytes [17]. It should be noted here that these studies used different treatment protocols with high- or low-dose MitoQ, short (minutes to hours) exposure times, and different cell types ranging from primary healthy cells to diseased or immortalized cells. In contrast to these previous studies, we here aimed to elucidate the effects of MitoQ on NRF2 signalling in human aortic SMCs in more detail. In particular, we asked to what extent NRF2 signalling is affected in aortic SMCs derived from AAA (AAA-SMC) and whether MitoQ can improve NRF2 signalling and reduce ROS production in these cells.

## 2. Materials and Methods

### 2.1. Patient and Control Tissue Samples

In total, 22 formalin-fixed and paraffin-embedded (FFPE) biopsies derived from AAA and nine control specimens derived from healthy donors were provided by the Vascular Biobank Heidelberg (VBBH). In addition, corresponding cryopreserved tissue samples were obtained from the VBBH for extraction of RNA and protein. Aortic wall specimens were obtained from patients who underwent open AAA repair between 2021 and 2023 at the department of Vascular and Endovascular Surgery, Heidelberg University Hospital (Germany). Before surgery, all patients gave their written informed consent to the study, which was approved by the ethical committee of the University of Heidelberg (S-301/2013 and amendments; S-673/2023). In addition, nine aortic artery samples (control group), free of macroscopic disease, were derived from organ transplantations from anonymous individuals, as previously described [18].

### 2.2. Immunohistochemical Staining

Processing and immunohistochemical staining were performed following standard procedures by using the following primary antibodies: rabbit-anti-human NRF2 (1:500; #PA5-27882, Invitrogen, Life technologies, Carlsbad, CA, USA); rabbit-anti-human KEAP1 (1:400; #bs-3648R, Bioss antibodies, Woburn, MA, USA); rabbit-anti-8-OHdG (1:800; #bs-1278R, Bioss antibodies, Woburn, MA, USA); mouse-anti-human SMA (1:1000; #M0851, Agilent DAKO, Santa Clara, CA, USA); and rabbit-anti-human CD45 (1:400; #13917, pan-leukocyte marker; Cell Signaling Technology, Inc., Danvers, MA, USA). Detailed staining protocols are available from the authors upon request. Briefly, 4 µm sections were deparaffinized, rehydrated, and incubated in 100 mM citrate buffer pH 6.0 for antigen retrieval, prior to incubation with the primary antibody at 4 °C overnight. After washing, detection was performed by using the Dako REAL Detection System Peroxidase/AEC rabbit/mouse (Dako; Agilent Technologies, Inc., Clostrup, Denmark), according to the recommendations of the manufacturer. All sections were counterstained with hematoxylin.

### 2.3. Imaging Analysis of Immunohistochemically Stained Samples

All slides were scanned using the NanoZoomer S60 from Hamamatsu, Hamamatsu City, Japan. Image analysis was performed using the open-source software QuPath-0.5.1. First, the entire area of all preparations (NRF2, KEAP1, 8-OHDG, αSMA, and CD45) was labelled using the wand tool. Then, taking into account the immunohistochemical staining against SMA, the media and intima were also labelled with the wand tool, whereby areas with strong inflammatory infiltration were specifically excluded based on a comparison with the immunohistochemical staining against CD45. The “Estimate stain vectors” function was then used for colour deconvolution (colour separation) to better separate the two colours of the preparations, the haematoxylin blue and the staining colour of the respective target protein. Using the “Pixel classification” function, a classification system with an appropriate threshold of 0.2 was established, which classifies each pixel of the specimen as positive or negative depending on the intensity of the DAB staining. To establish this, a small representative section was selected, and the parameters of the pixel classifier were gradually adjusted so that the areas recognized by it as positive correspond to the areas that are also to be evaluated as positive. This classification system was then used to measure the surface area of the previously defined regions. This made it possible to determine the proportion of NRF2, KEAP1, CD45, and 8-OHDG-positive areas in relation to the total area, both for the entire specimen and for the media and intima regions.

### 2.4. Extraction of RNA from Cryopreserved Human Tissue Samples

For extraction of RNA and proteins, the cryopreserved human tissue samples were ground under liquid nitrogen. The resulting tissue powder was then aliquoted and frozen at −80 °C until further analysis. For RNA extraction, 30–200 mg of tissue powder was mixed with 600 μL of RLT buffer containing mercaptoethanol (1:100 dilution) in a 2 mL Eppendorf tube. Following the protocol of the RNeasy Mini Handbook (Qiagen, Hilden, Germany; June 2023 version: Purification of total RNA from Animal Tissues, step 3b), the tissue lysate was further homogenized using ultrasonic pulses and QIA shredder columns. After centrifugation for 2 min at 14,000 *g*, the column was discarded, and the flow-through, representing the pure lysate, was centrifuged again for 2 min at 14,000 *g*. RNA extraction was performed using the RNeasy Fibrous Tissue Mini Kit (Qiagen, Hilden, Germany), following the instructions of the manufacturer.

### 2.5. Quantitative Reverse Transcriptase-Polymerase Chain Reaction (RT-qPCR)

RNA extracts were converted to cDNA using SuperScript III reverse transcriptase and Oligo-dT primers, as described previously [19]. For real-time PCR, the Biozym qPCR Kit Separate ROX (Biozym Scientific GmbH, Hessisch Oldendorf, Germany) was added to the diluted cDNA samples and appropriate primers (Supplementary Table S1). Samples were loaded onto 96-well plates and analysed in an ABI StepOne Plus thermocycler (ThermoFisher Scientific, Waltham, MA, USA). Quantitative analysis of gene expression was performed relative to the mean expression of *GAPDH* and *ACTB* in corresponding samples by using the 2^−ΔΔCt^ method [20].

### 2.6. Extraction of Nuclear and Cytoplasmic Protein from Cryopreserved Human Tissue Samples

Extraction of nuclear and cytoplasmic protein from tissue samples was performed by using a nuclear extraction kit (Abcam, Cambridge, UK). Briefly, 100 mg of the ground tissue powder was homogenized with 500 μL of the 1X extraction buffer (provided in the kit) using 50 strokes with a Douncer homogenizer (Carl Roth GmbH, Karlsruhe, Germany), followed by 15 min of incubation on ice and subsequent centrifugation. The supernatant, containing the cytoplasmic proteins, was removed and frozen at −80 °C until further use. The pellet was further used for extraction of nuclear proteins, following the instructions of the manufacturer. The final supernatant, containing the dissolved nuclear proteins, was removed and frozen at −80 °C until further analysis. The concentration of both the cytoplasmic and nuclear protein extracts was determined colorimetrically using the Pierce BCA Protein Assay Kit (ThermoFisher Scientific, Waltham, MA, USA).

### 2.7. Western Blotting

Protein lysates were thawed on ice, and 25 µg of each sample was separated by SDS-PAGE using 12% Mini-Protean TGX Gels (BIORAD, Hercules, CA, USA). Each gel was additionally loaded with 10 µL NRF2 control cell extracts (total cell extracts of A172 +/− MG132; # 41645; Cell Signaling Technologies, Danvers, MA, USA) to assign the correct bands. After SDS-PAGE, gels were transferred to nitrocellulose membranes using the “wet transfer” protocol with transfer buffer (25 mM tris, 192 mM glycin, 0.1% SDS, and 20% methanol) at 100 V for 45 min and cooling. After washing with TBST (tris-buffered saline, 1% Tween 20) and blocking (5% skim milk in TBST), the blots were incubated overnight with the primary antibody (NRF2 (D1Z9C) rabbit monoclonal antibody #12721; dilution 1:1000 in 5% skim milk/TBST; Cell Signaling Technologies, Danvers, MA, USA). Following three washing steps in TBST, the blots were exposed to the secondary antibody (anti-rabbit IgG-HRP-inked antibody; #7074; dilution 1:2000 in 5% skim milk/TBST; Cell Signaling Technologies, Danvers, MA, USA) for 1 h at room temperature. After three more washing steps in TBST, blots were covered with ECL (Western Bright ECL, Advansta, San Jose, CA, USA), and the signals were visualized using an imaging system (Vilber Lourmat, Eberhardzell, Germany). For the blots containing nuclear extracts, Histon H3 (Histon H3 (D1H2) XP rabbit mAb; #4499; Cell Signaling Technologies, Danvers, MA, USA) was used as a reference. For the blots containing cytoplasmic extracts, beta-Actin (β-Actin (13E5) rabbit mAb; #4970; Cell Signaling Technologies, Danvers, MA, USA) was used as a reference. ImageJ/FIJI (1.53q, https://imagej.net/ij/ (64 bit)) was used for quantification of signals corresponding to NRF2. Briefly, original digital images were converted to an 8-bit grayscale. Brightness was adjusted to non-saturated pixels, and density profiles were captured with the gel analyzer tool, measuring peak areas. Background noise was subtracted, and values were normalized against a loading control using a spreadsheet.

### 2.8. Human Primary and Immortalized Aortic Smooth Muscle Cells

AAA-SMCs were isolated from human AAA biopsies that were obtained during open surgery. All procedures were performed according to the standard operating procedures of the Vascular Biobank Heidelberg (VBBH), as previously described [21]. Donors were tested for viral infections (HIV, HBV, and HCV) on a routine basis by the central laboratory of the University Hospital Heidelberg. Only tissues from patients who tested negative for these viruses were used for isolation of cells. A total of *n* = 4 primary AAA-SMC cultures were included. In addition, human aortic smooth muscle cells (healthy VSMC) derived from healthy donors (*n* = 4) were purchased from PromoCell (Heidelberg, Germany). The immortalized VSMC line (iHAoSMC) was obtained from InnoProt (P10456-IM, Derio, Spain).

### 2.9. Vascular Smooth Muscle Cell Culture

AAA-SMC and healthy VSMC were grown in Smooth Muscle Cell Growth Medium 2 (SMC-GM2, PromoCell, Heidelberg, Germany) supplemented with 0.05 mL/mL fetal calf serum, 0.5 ng/mL epidermal growth factor, 2 ng/mL basic fibroblast growth factor, and 5 µg/mL insulin (all provided by the manufacturer). The iHAoSCM was grown in Smooth Muscle Cell Medium-Plus (SMCM-Plus, InnoProt, Derio, Spain). All cells were grown without antibiotics at 37 °C and 5% CO_2_ in a humidified atmosphere. For subculturing, the primary cells were split 1:2 to 1:4 when reaching 80–90% confluence. The iHAoSMC were split 1:8 to 1:10. Primary cells were used at passages 5 to 10 for the experiments in this study. Because of the limited growth of primary cells, some experiments were performed with only part of the cell lines. The precise number of cell lines used for each experiment is described in the figure legends. Details of the cell cultures are described in Supplementary Table S2.

### 2.10. Treatment of the Cells

For the dose-response experiment involving induction of acute oxidative stress, cells were seeded in 96-well plates and treated with 0–500 µM H_2_O_2_ (Merck/Sigma-Aldrich, Darmstadt, Germany) for 30 min before the replacement of the medium and cells were allowed to recover for 0 h, 2 h, 4 h, or 24 h. For assessing the protective effects of MitoQ (mitoquinone mesylate, Biorbyt Ltd., Cambridge, UK), cells were incubated with 10 nM MitoQ for 7 days. The first 6 days of incubation took place in the cell culture bottle, with the medium being changed to MitoQ-containing medium on days 1 and 3. On day 6, the cells were counted and plated at the desired concentration in new MitoQ-containing medium in 96-well plates or 6-well plates for another 24 h, before 100 µM H_2_O_2_ was added for 30 min. The NRF2-inhibitor ML385 ((Biozol Diagnostica, Eching, Germany) final concentration: 1 µM) was added 2 h before induction of oxidative stress by H_2_O_2_, as indicated in the figures. For regeneration, the medium was replaced again with SMC-GM2 or SMCM-Plus without MitoQ for the duration specified in the figure legends.

### 2.11. Cell Viability Assay

The WST-1 cell proliferation reagent (Merck, Darmstadt, Germany) was used for analysis, as described by the manufacturer. Briefly, cells were grown overnight in 96-well plates (+/− MitoQ) at a density of 1 × 10^4^ cells/well in 100 µL SMC-GM2 with reduced serum (1% supplement) to synchronize the cell cycle. Assays were initiated by changing the medium to complete SMC-GM2 containing different test reagents, as described in the figures. For analysis, 10 µL of WST-1 proliferation reagent was added to each well, and absorbance was measured at 450 nm versus 620 nm with a TECAN Spark reader, as recommended by the manufacturer. To determine the relative proliferation rate, all data (A450–A620) were normalized to the absorbance at the start. All experiments were performed in triplicate.

### 2.12. Cell Lysis and Preparation of Total Protein Extracts

Cells were seeded and treated in 6-well plates, as described in the figure legends. Cells were washed in ice-cold phosphate-buffered saline (PBS) and harvested using a cell detach kit (PromoCell, Heidelberg, Germany). The cell suspension was centrifuged, and the pellet was lysed in cell extraction buffer (FNN0011, Invitrogen/Life Technologies, Carlsbad, CA, USA), supplemented with 1 mM PMSF for 30 min on ice, with vortexing at 10-min intervals. The extracts were transferred to microcentrifuge tubes and centrifuged at 13,000 RPM for 10 min at 4 °C. Lysates were stored in aliquots at −80 °C until further use. For analysis of protein concentrations, samples were thawed, and the total protein concentration of each sample was determined using a BCA assay (Pierce BCA protein assay kit, ThermoFisher Scientific) before performing assays.

### 2.13. Extraction of Nuclear Protein from Treated Cells

After treatment, cells were harvested using a cell detach kit (PromoCell, Heidelberg, Germany), transferred to microcentrifuge tubes, and centrifuged. For preparation of nuclear extracts, the nuclear extraction kit (Abcam, ab113474, Abcam, UK) was used following the instructions of the manufacturer. Briefly, the pellet was resuspended in 300 μL 1× pre-extraction buffer with DTT and PIC (provided in the kit) and incubated on ice for 10 min. The suspension was homogenized for 10 s by vortexing and centrifuged for 1 min at 12,000 RPM to separate the cytoplasmic fraction from the cell nuclei. The pellet, containing the nuclei, was further processed according to the instructions of the manufacturer, and nuclear extracts were frozen at −80 °C until further usage for analyzing NRF2 activity.

### 2.14. Quantification of NRF2 and HO-1 Protein Concentrations by ELISA

The Human NRF2 ELISA Kit and Human Heme oxygenase 1 ELISA Kit (both from Invitrogen/Life Technologies, Carlsbad, CA, USA) were used for analysis, following the instructions of the manufacturers. Cell lysates were diluted 1:20 to 1:40 and analysed in duplicates. Absorption was measured using the TECAN Spark spectrophotometer. The determined NRF2 and HO-1 protein concentrations were normalized to the respective total protein concentrations of the samples.

### 2.15. Measurement of NRF2 Binding Activity to DNA (NRF2 Activity Assay)

Nuclear extracts were thawed, and the protein concentration was determined by using a Bradford assay (Bio-Rad protein assay kit, Bio-Rad laboratories, Hercules, CA, USA). NRF2 binding activity to DNA was measured by using the NRF2 transcription factor assay kit (Cayman Chemical, Ann Arbor, MI, USA) following the instructions of the manufacturer. Briefly, 10 µL of the nuclear extracts were plated on a 96-well plate, precoated with NRF2-DNA target sequence. After incubation and washing, a primary antibody (anti-NRF2 Ab) was added, followed by washing and addition of the secondary antibody (goat-anti rabbit HRP Ab, all provided in the kit). After addition of the developing reagent and stop solution, absorbance was read at 450 nm using a TECAN Spark spectrophotometer (TECAN, Männedorf, Switzerland). Samples were analysed in duplicates, and data were normalized to the total protein concentration of the nuclear extracts.

### 2.16. RNA Extraction from Cultured Cells and Quantification of Gene Expression by RT-qPCR

Cells were seeded and treated in 6-well plates as described in the figure legends. RNA extraction was performed by using the RNeasy Mini Kit or QIAwave RNA Mini Kit as described by the manufacturer. Briefly, cells were washed with PBS and harvested in RLT buffer (provided in the kit), and the lysate was stored at −80 °C until further use. The final extraction was performed following the manufacturer’s protocol “Purification of total RNA from animal cells using spin technology.” RNA concentrations were determined using the NanoDrop 2000c spectral photometer (ThermoFisher Scientific, Waltham, MA, USA), and RNA samples were again stored at −80 °C until further use. Prior to cDNA synthesis, 0.5 µg of RNA from each sample was treated with DNase 1 in DNase 1 buffer (Invitrogen/Life Technologies, Carlsbad, CA, USA) for 15 min to remove any remaining DNA residues. After inactivation of the DNase by adding EDTA and incubation for 10 min at 60 °C, the RNA was reverse transcribed using oligo-dT primers and SuperScript III reverse transcriptase (Invitrogen/Life Technologies, Carlsbad, CA, USA) following the instructions of the manufacturer. Real-time PCR was performed as described above.

### 2.17. Analysis of ROS Production

Cells were pretreated with 10 mM MitoQ or vehicle for 7 days and subsequently transferred to 96-well microtiter plates at a density of 10,000 cells/well the day before the experiment. Cells were then treated with different compounds (H_2_O_2_, NRF2 inhibitor ML385) under the desired conditions. For analysis of ROS production, CellROX green oxidative stress reagent (Invitrogen/Life Technologies, Carlsbad, CA, USA) was used as recommended by the manufacturer. Briefly, CellROX green reagent was added to the cells at a final concentration of 5 µM without changing the cell culture medium and incubated for 30 min at 37 °C. For counterstaining of the nuclei, NucBlue Live Ready Probes Reagent (Hoechst 33342) was added simultaneously. Medium was removed, and the cells were washed three times in PBS. Fluorescence was analysed in two different ways. A Keyence fluorescence microscope was used for taking photographs. For analysis, fluorescence was quantified using a TECAN infinite F200 PRO reader (TECAN, Männedorf, Switzerland). Green fluorescence intensity was normalized to the blue fluorescence intensity to achieve relative ROS production.

### 2.18. Statistical Analysis

The statistical analysis of the data and the creation of graphs were performed using Graphpad Prism Software, Version 10.6.1. The data was first tested for normal distribution in order to select appropriate statistical tests. The respective statistical tests used in each case are listed in the legends of the figures.

## 3. Results

### 3.1. Abundance of KEAP1, NRF2, and 8-OHdG in the Media and Intima of AAA Tissue Samples and Healthy Aorta

**Figure 1:**
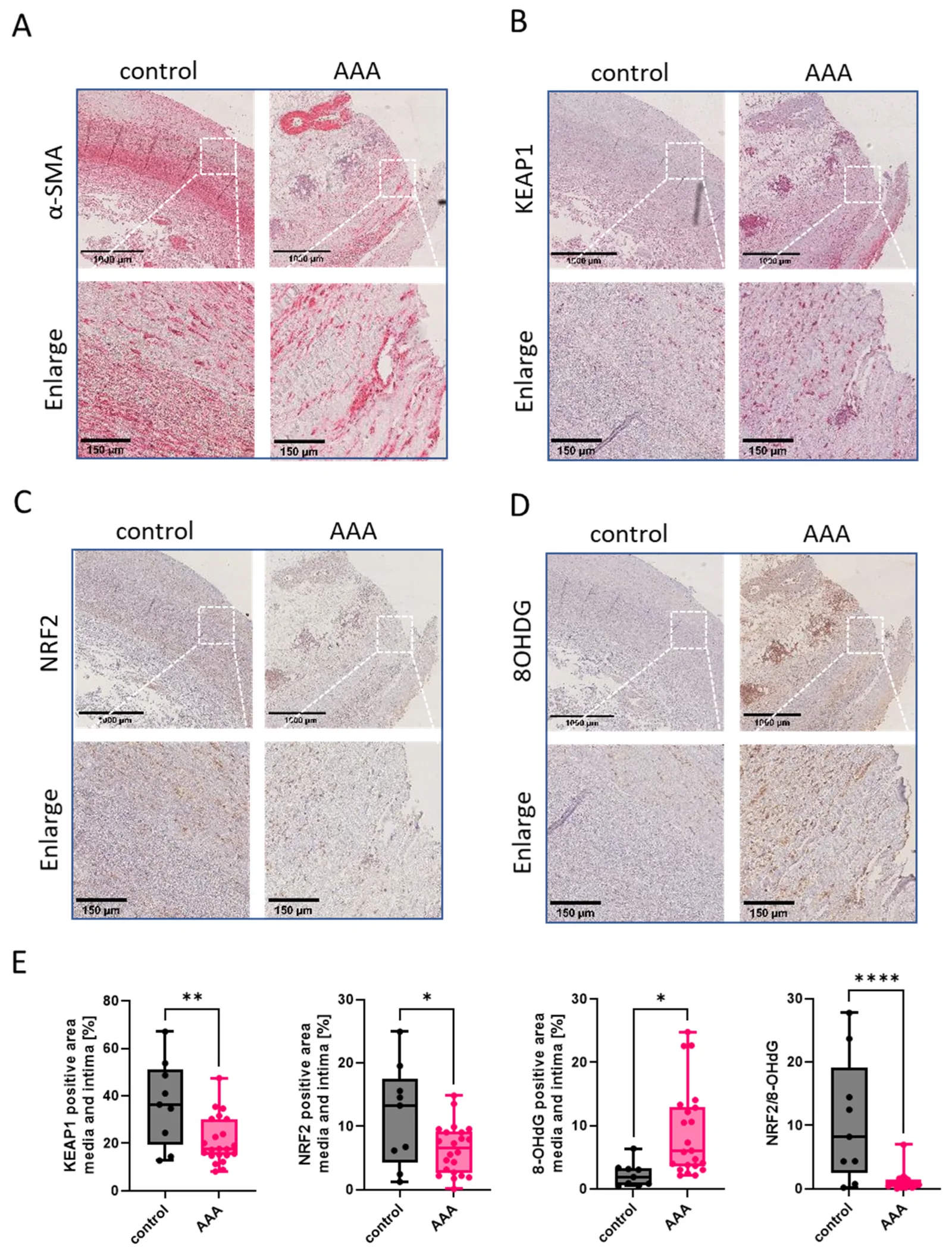
Immunohistochemical analysis of αSMA, KEAP1, NRF2, and 8-OHdG in the media and intima of AAA tissue samples and healthy aorta (control). (**A**–**D**) Representative image details of *n* = 22 AAA and *n* = 9 healthy control tissue samples are shown. The white dashed lines and squares indicate the enlarged sections. (**E**) The entire available surface area of each specimen was used for the analysis. Box plots show the median, maximum, and minimum of percent positive staining normalized to the total media/intima area. Each colour circle represents a normalized value of an individual tissue sample. Data were statistically analysed by unpaired *t* test. *: *p* < 0.05, **: *p* < 0.01, ****: *p* < 0.0001. 1000 μm, 150 μm.

We first wanted to compare NRF2 levels in situ in human-healthy versus aneurysmal aortic tissue. Immunohistochemistry was used to compare the relative levels of NRF2 and its inhibitor KEAP1 in the tunica media and intima of AAA tissues and healthy aortic tissues (controls). The tunica media and tunica intima were defined by the presence of alpha-smooth muscle actin (αSMC), collagen, and elastic fibres. Immune cells were defined by positive staining for CD45. As expected, the proportion of VSMCs (αSMA-positive cells) was significantly reduced in the media/intima of AAA tissues (Figure 1A and Supplemental Figure S1A), whereas the proportion of immune cells (CD45-positive cells) was significantly increased (Supplemental Figure S1B).

Both KEAP1 and NRF2 were detectable in large quantities in the tunica media and intima of healthy aortic samples, but were minimally abundant in these areas of AAA samples (Figure 1B,C).

In contrast, 8-OHdG, a marker for oxidative stress, was particularly detected in AAA samples (Figure 1D). Quantitative analysis revealed a significant difference in NRF2 or KEAP1 positive areas between AAA samples and healthy controls, and the ratio between NRF2-positive areas and 8-OHdG-positive areas was significantly reduced in AAA samples, indicating alterations in the NRF2-mediated response to oxidative stress (Figure 1E, Supplemental Figure S1C). Relative NRF2 abundance in VSMCs (NRF2/αSMA-positive areas) of AAA tissues was slightly increased without differing significantly between AAA and healthy aortic tissue (Supplemental Figure S1D).

### 3.2. Expression of NRF2 and Its Target Genes in Whole Tissue Extracts of AAA Tissue Samples and Healthy Aorta

**Figure 2:**
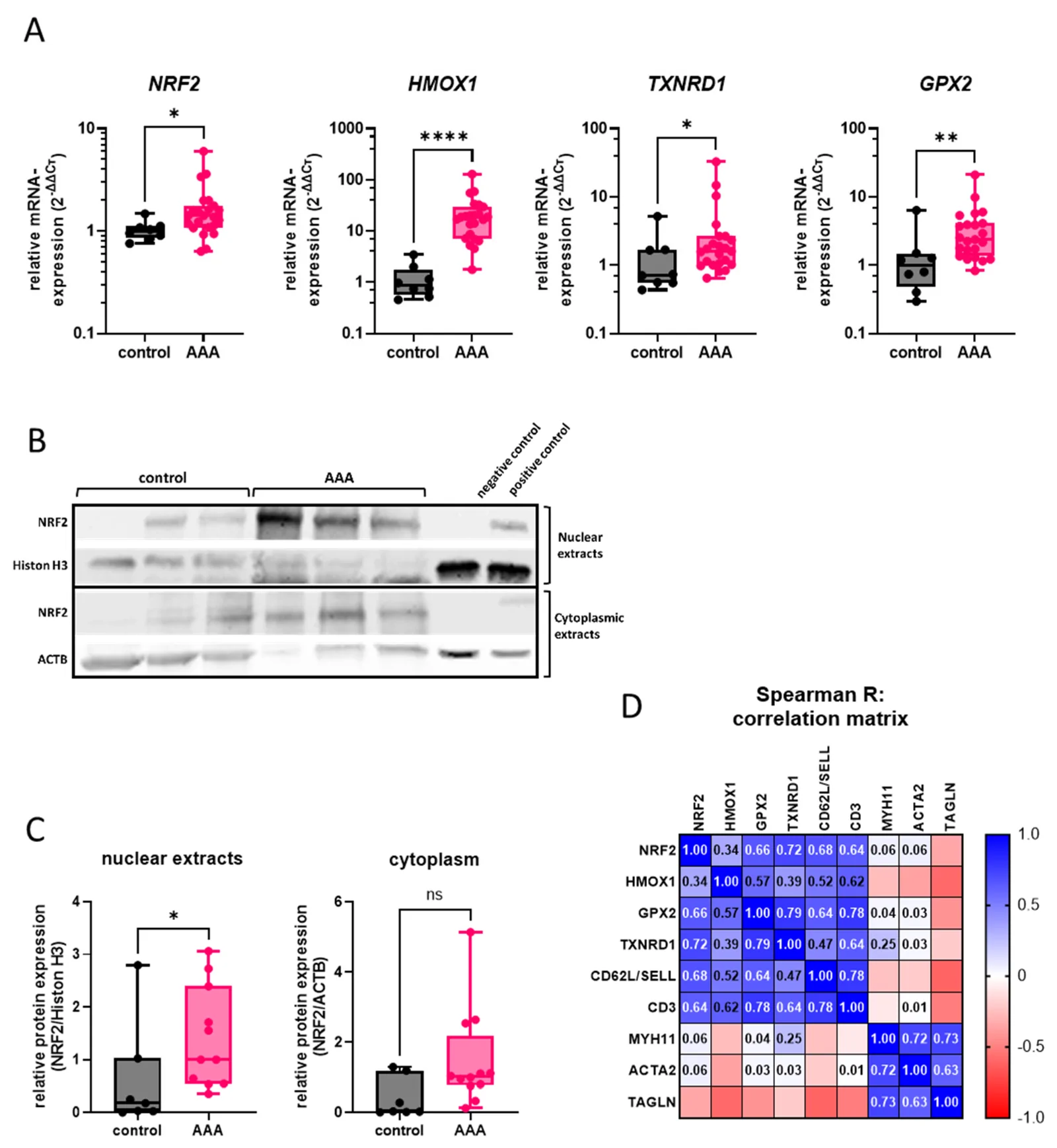
Quantification of NRF2 transcript and protein expression from whole tissue extracts. (**A**) Relative transcript expression of NRF2 and its target genes HMOX1, TXNRD1, and GPX2. Box plots show the median, maximum, and minimum of 2^−ΔΔCt^ values derived from real-time qPCR. Each dot represents the mean value of an individual sample. (**B**) Western blot showing NRF2 protein expression in nuclear and cytoplasmic extracts of three representative AAA and three control samples. (**C**) Quantification of the Western blot analysis (*n* = 7 control and *n* = 11 AAA tissue extracts). Box plots show the median, maximum, and minimum of the relative protein expression after normalization as described in the methods section. Each colour circle represents the value of an individual sample (**A**,**C**) Data were analysed by Mann–Whitney test. *: *p* < 0.05, **: *p* < 0.01, ****: *p* < 0.0001, ns: not significant. (**D**) Correlation matrix visualizing the relationships between transcript expression of different genes in whole tissue extracts.

Analysis of whole tissue extracts derived from cryopreserved samples revealed significantly increased transcript expression of *NRF2* (*NFE2L2*) and three of its target genes in AAA samples compared to controls (Figure 2A). Similarly, NRF2 protein expression was significantly higher in AAA tissue extracts compared to controls (Figure 2B,C). To determine whether the increased NRF2 expression in whole tissue extracts was due to VSMCs or immune cells, the extracts were tested for the expression of VSMC-specific and immune cell-specific markers (Supplemental Figure S2). Further analysis revealed a strong correlation of *NRF2* and its target genes with markers for immune cells (CD3: marker for T cells; CD62L: marker for naïve lymphocytes, neutrophils, and monocytes) but not with markers for VSMC (Figure 2D and Supplemental Figure S2F–G). This indicates that the increased NRF2 expression detected in whole cell lysates of AAA samples was mainly attributable to immune cells, which means that the exact proportion of NRF2 in VSMCs cannot be precisely determined.

### 3.3. Comparison of Baseline NRF2 Protein Levels, NFE2L2 Transcript Expression, and NRF2 Activity in Healthy VSMC, AAA-SMC, and iHAoSMC

**Figure 3:**
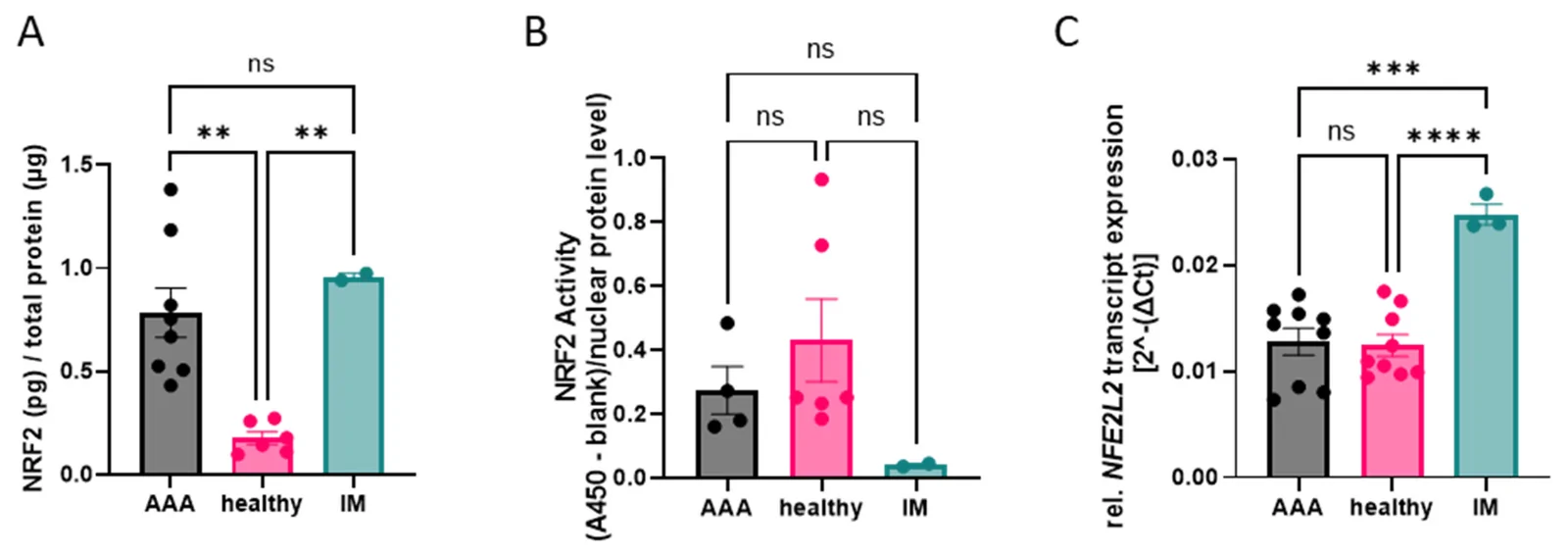
Comparison of baseline NFR2 protein levels (**A**) NFR2 activity (**B**) and NRF2 transcript expression without additional oxidative stress induction. (**A**) NRF2 protein levels were determined by ELISA and normalized to the total protein level of each sample. Data are shown as pg NRF2 per µg protein. Each color circle represents a normalized value derived from *n* = 4 AAA-SMC, *n* = 3 healthy VSMC samples and one immortalized cell line (IM), analysed in duplicate. (**B**) NRF2 binding activity to DNA was assayed from nuclear extracts by using a colorimetric NRF2 activity assay. Data were normalized to the protein content of the corresponding nuclear extracts. Each color circle represents a normalized value derived from *n* = 2 AAA-SMC, *n* = 3 healthy VSMC and one immortalized cell line (IM), analysed in duplicate. (**C**) NFE2L2 transcript expression was analysed by qPCR using specific primers. Data show the 2^−(ΔCt)^ values. Each colour circle represents a normalized value of *n* = 2 AAA-SMC (AAA), *n* = 3 healthy VSMC (healthy), and one immortalized cell line (IM), analysed in triplicate. Bars represent the mean ± SEM. Data were statistically analysed by ordinary one-way ANOVA and Tukey’s multiple comparison test; ns: not significant. ** *p* < 0.01, *** *p* < 0.001, **** *p* < 0.0001.

To investigate the functionality of NRF2 expressed in VSMC in more detail, we used in vitro cultures of isolated AAA-SMC, healthy VSMC and iHAoSMC for comparison of NRF2 protein levels, *NFEL2L* transcript expression, and NRF2 binding activity to the antioxidative response elements (ARE). The baseline NRF2 protein level was significantly higher in AAA-SMC and in iHAoSMC than in the healthy VSMC (Figure 3A and Supplemental Figure S3A). In contrast, NRF2 binding activity to DNA was similar in AAA-SMC and in healthy VSMC but slightly reduced in iHAoSMC (Figure 3B and Supplemental Figure S3B). No difference was detected for the *NFEL2L* transcript expression levels between AAA-SMC and healthy VSMC, whereas it was significantly higher in iHAoSMC (Figure 3C). Together, this indicates a significantly increased stability of the NRF2 protein in AAA-SMC, despite moderate activity. Expression and protein levels of the NRF2 adaptor KEAP1 were heterogeneous and did not correlate significantly with NRF2 protein levels (Supplemental Figure S3C–E).

### 3.4. H_2_O_2_ Differentially Affects Cell Viability in Healthy VSMC, AAA-SMC, and iHAoSMC

**Figure 4:**
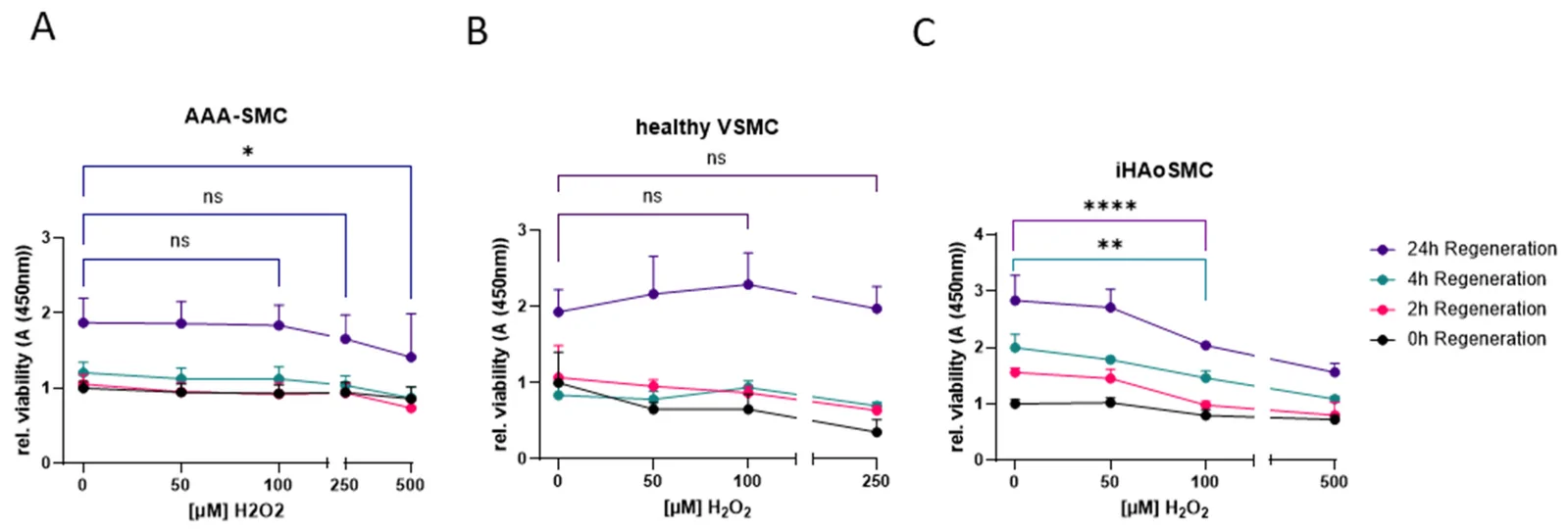
Dose response of AAA-SMC (**A**), healthy VSMC (**B**), and iHAoSMC (**C**) to acute oxidative stress induced by H_2_O_2_. Cells were exposed to different concentrations of H_2_O_2_ for 30 min and analysed for viability using a WST-1 viability assay after recovery times of 0 h, 2 h, 4 h, and 24 h in smooth muscle cell growth medium without H_2_O_2_. Data were statistically analysed by two-way ANOVA and Tukey’s multiple comparison test; ns: not significant, *: *p* < 0.05, ** *p* < 0.01, ****: *p* < 0.0001.

**Figure 5:**
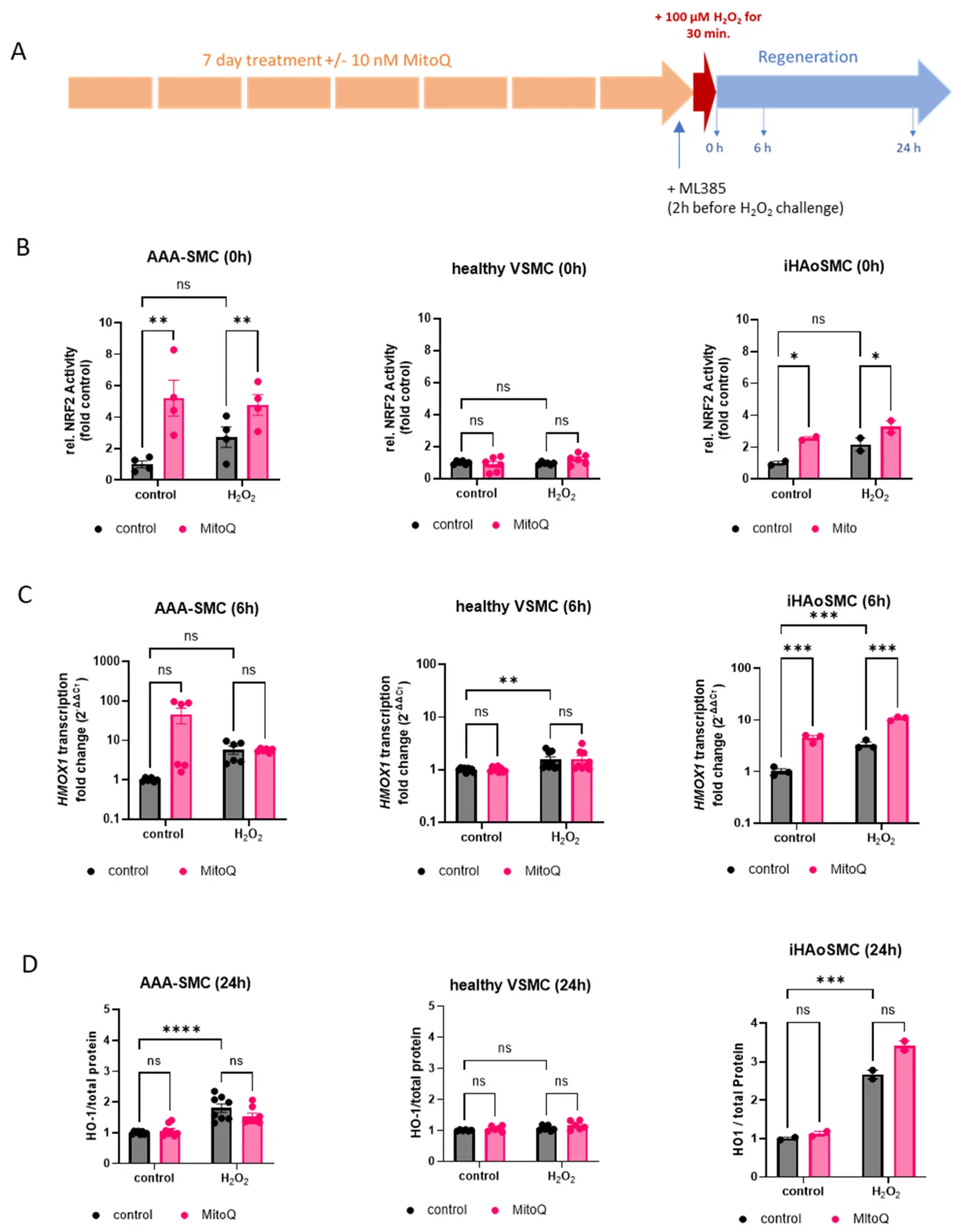
Response of AAA-SMC, immortalized iHAoSMC, and healthy VSMC against acute oxidative stress induced by exposure to H_2_O_2_. (**A**) Treatment scheme for induction of acute oxidative stress. Cell lines were treated with 10 nM of MitoQ or vehicle for 7 days before they were challenged with 100 µM H_2_O_2_ for 30 min. (**B**) NRF2 binding activity to DNA (antioxidative response element, ARE). Nuclear extracts were harvested immediately after H_2_O_2_ treatment and analysed by using an NRF2 activity assay. (**C**) Transcriptional expression of the NRF2 target gene HMOX1 (HO1), 6 h after H_2_O_2_ treatment. Data show the 2^−ΔΔCt^ values derived from qPCR. (**D**) HO1 protein expression was analysed by ELISA. Protein extracts were harvested after 24 h of regeneration. (**B**–**D**) Data are derived from *n* = 2 individual AAA-SMC cultures, *n* = 3 individual healthy VSMC cell cultures, and one iHAoSMC line. Bars represent the mean ± SEM. Data were statistically analysed by two-way ANOVA and Tukey’s multiple comparison test; ns: not significant, *: *p* < 0.05, ** *p* < 0.01, ***: *p* < 0.001, ****: *p* < 0.0001.

To better elucidate the response to oxidative stress of SMCs derived from different origins, we first compared the viability of primary AAA-SMC, isolated from patients, with healthy VSMC, and the cell line iHAoSMC in response to increasing doses of H_2_O_2_. AAA-SMC proved to be surprisingly robust against acute oxidative stress caused by a 30-min exposure to H_2_O_2_. Doses up to 100 µM did not affect cell viability. After the H_2_O_2_ challenge and 24 h regeneration time, the cell number was slightly reduced in the group that was exposed to 500 µM H_2_O_2_ (Figure 4A). Healthy VSMC could fully recover from H_2_O_2_ concentrations up to 250 µM within 24 h, without significant reduction of cell viability after exposure to 100 and 250 µM H_2_O_2_ concentrations and 0, 2, and 4 h of recovery (Figure 4B). In contrast, iHAoSMC appeared to be more vulnerable, showing a significantly reduced viability 2 h, 4 h, and 24 h after the H_2_O_2_ challenge using 100 µM or higher concentrations for induction of oxidative stress (Figure 4C). There was no full recovery of iHAoSMC exposed to H_2_O_2_ concentrations above 100 µM even after 24 h of regeneration. Chronic oxidative stress (for 1, 2, 4, and 24 h) caused by H_2_O_2_ in iHAoSMC led to a substantial reduction in cell viability after exposure to H_2_O_2_ concentrations above 500 µM (Supplemental Figure S4). Based on these preliminary results, a 30-min exposure of 100 µM H_2_O_2_ with subsequent regeneration times was selected for further experiments to analyse acute oxidative stress response (Figure 5A).

### 3.5. MitoQ Has Diverse Effects on Viability, Apoptosis Rate, and Cell Growth in Aortic VSMC of Different Origins

To analyse whether MitoQ can alleviate acute oxidative stress in aortic VSMC, we first investigated the dose-response relationship of MitoQ in VSMC cells of different origins. As shown in Supplemental Figure S5A, MitoQ can exert cytotoxic effects on VSMC. Interestingly, cell viability appeared to be more severely impaired by MitoQ in healthy VSMC than in AAA-SMC. In healthy VSMC, the half-maximal inhibitory concentration (IC50) was 1.228, 1.041, and 0.991 µM after 24 h, 48 h, and 72 h, respectively. In contrast, in AAA-SMC, the IC50 was more than 10-fold higher with 26.908 µM, 12.389 µM, and 10.106 µM after 24 h, 48 h, and 72 h, respectively.

To better understand the mechanism of cytotoxicity by MitoQ, we analysed the apoptosis profile by performing a caspase-3/7 activity assay following drug exposure. The number of apoptotic cells in healthy VSMC increased with time, but not with increasing MitoQ concentrations, whereas the amount of apoptotic AAA-SMC was not significantly affected by MitoQ, even after 72 h of exposure to 1 µM concentrations (Supplemental Figure S5B). This indicates that apoptosis may not be the major mechanism responsible for reduction of cell viability. To investigate the anti-proliferative activity of long-term MitoQ treatment (7 days), we finally determined the cell numbers after drug exposure. Independent of the cell origin, the VSMC numbers were significantly reduced by exposure to MitoQ at doses above 100 nM and 1 µM (Supplemental Figure S5C).

Based on these results and because we wanted to mimic physiological conditions of MitoQ supplementation in humans, where a low-, nontoxic dose is given for a longer time, we chose a concentration of 10 nM MitoQ in combination with 7 days of exposure for the following in vitro experiments. Moreover, to avoid direct interaction of MitoQ and H_2_O_2_, the cell culture medium containing MitoQ was removed after the 7-day treatment and replaced by H_2_O_2_ -containing medium for 30 min. The medium was replaced again, and the response was measured at different time points following the acute oxidative stress test. Figure 5A shows the treatment scheme for induction of oxidative stress in cells pretreated with MitoQ (10 nM for 7 days) versus vehicle-treated cells.

### 3.6. MitoQ Increases NRF2 Binding Activity to Its DNA Response Element (ARE) in AAA-SMC and in iHAoSMC but Not in Healthy VSMC

We examined the potential positive effects of MitoQ on NRF2 signalling in VSMC derived from different origins exposed to acute oxidative stress. As shown in Figure 5B, the binding activity of NRF2 to its DNA response element (ARE) was slightly increased in response to acute oxidative stress by H_2_O_2_ in AAA-SMC and in iHAoSMC, but not in healthy VSMC. Moreover, NRF2 activity was significantly higher in AAA-SMC pretreated with MitoQ compared to cells grown without MitoQ. This applies both to control cells without acute oxidative stress and to cells that have been exposed to H_2_O_2_. The same reaction was observed in iHAoSMC, but not in healthy VSMC. The effects on NRF2 activity were confirmed in part by increased transcription of the NRF2 target gene HMOX1 in MitoQ-pretreated AAA-SMC and in iHAoSMC, but not in healthy VSMC (Figure 5C). Similar effects were observed on the expression of the NRF2 target genes SOD2 and TXNRD1 (Supplemental Figure S6A,B). Interestingly, expression of the NRF2 transcript NFEL2L was also increased in MitoQ-pretreated AAA-SMC in response to the H_2_O_2_ challenge compared to vehicle-treated cells, which suggests that MitoQ also exerts an effect on signalling upstream of NRF2 gene expression. There was no effect of MitoQ pre-treatment on the protein level of heme-oxygenase-1 protein (HO-1) at 24 h after the acute H_2_O_2_ challenge (Figure 5D).

### 3.7. MitoQ Reduces ROS Production in Response to H_2_O_2_ in AAA-SMC but Has No Effect on Healthy VSMC and iHAoSMC

**Figure 6:**
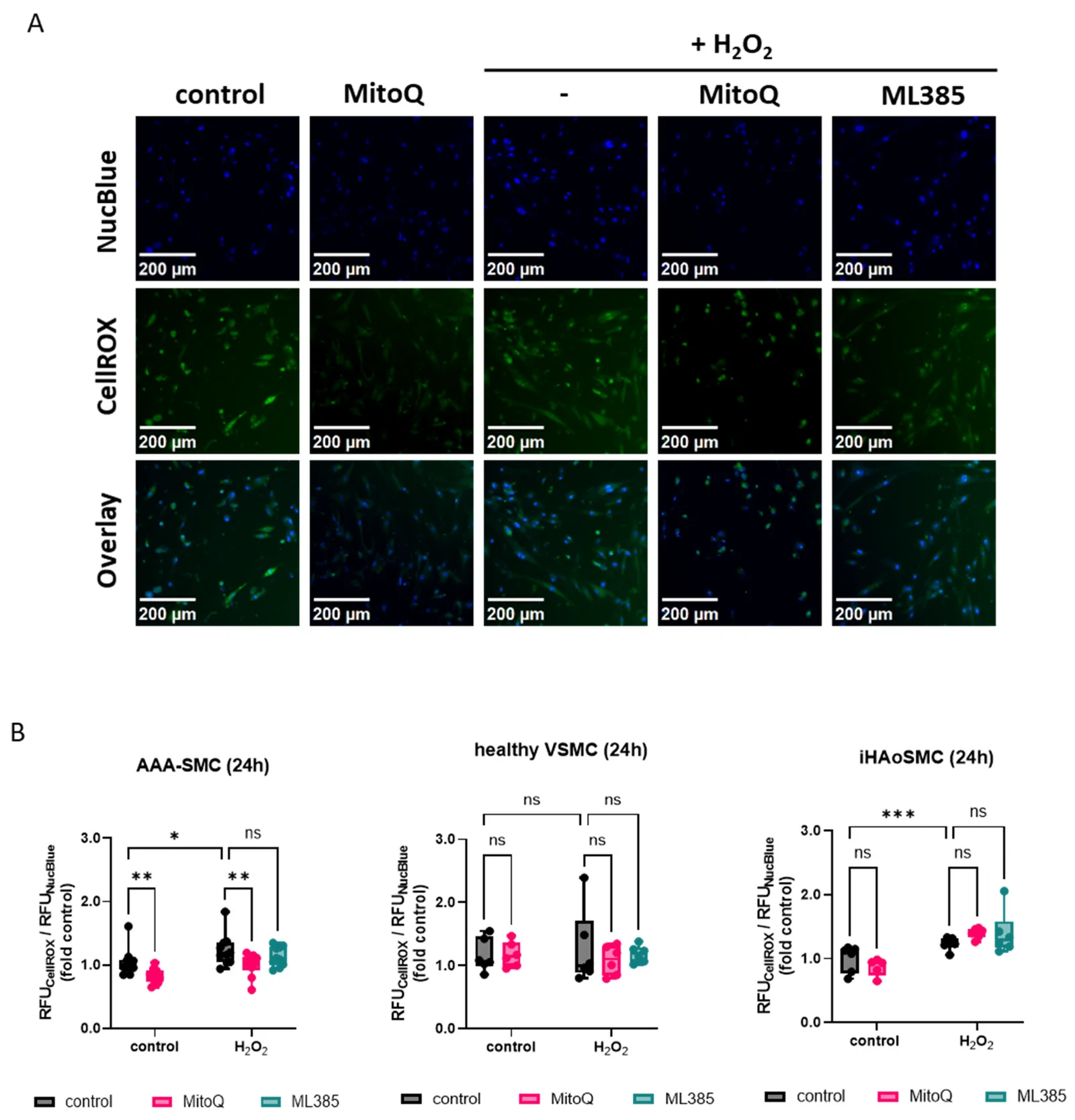
ROS production in AAA-SMC, immortalized iHAoSMC, and in healthy VSMC. Cells were exposed to acute oxidative stress by H_2_O_2_ for 30 min, and images were taken after 24 h of regeneration. (**A**) Representative images of ROS staining from a AAA-SMC line. ROS production was examined via CellROX fluorescent dye; nuclei were stained with NucBlue. (**B**) Quantification of ROS fluorescence intensity, normalized to NucBlue fluorescence. Data are derived from *n* = 2 individual AAA-SMC cultures analysed fivefold (**left panel**) from *n* = 3 individual healthy VSMC cell cultures analysed in triplicate (**middle panel**) and an iHAoSMC line analysed in six technical replicas (**right panel**). Box plots show the median, maximum, and minimum of values derived from the ROS assay. Each colour circle represents an individual value after normalization. Data were statistically analysed by two-way ANOVA and Tukey’s multiple comparison test; ns: not significant, *: *p* < 0.05, ** *p* < 0.01, ***: *p* < 0.001.

To investigate whether MitoQ pre-treatment alters ROS production, cells were stained with CellROX, an indicator of intracellular oxidative stress. In AAA-SMC, induction of acute oxidative stress by H_2_O_2_ further increased intracellular ROS production, which was significantly reduced in MitoQ-pretreated cells (Figure 6A,B). The effect was blunted by the NRF2-inhibitor ML385, indicating that MitoQ reduced ROS production via activation of NRF2. ROS production was likewise increased in iHAOSMC by H_2_O_2_, but there was no reduction of ROS production in MitoQ-pretreated iHAoSMC. Finally, there was no change in ROS production in healthy cells, neither in response to H_2_O_2_ nor by pre-treatment with MitoQ (Figure 6B).

## 4. Discussion

Previous studies on NRF2 signalling in AAA have been predominantly performed by using animal models and bioinformatic approaches from single-cell RNA sequencing data [9,10]. However, data from immunohistochemical analysis of the NRF2 expression and oxidative stress directly in human aneurysm samples are missing. This study investigated, for the first time, the distribution and expression of the antioxidant transcription factor NRF2, a major regulator of the antioxidative stress response, [9] and its inhibitor KEAP1, as well as their effects on oxidative stress in human aortic tissue and abdominal aortic aneurysms derived from surgeries. On the one hand, a reduced expression of NRF2 and KEAP1 was observed in the intima and media, accompanied by an increase in the marker for oxidative stress, 8-OHdG. The reduced NRF2 levels were primarily attributable to the massive loss of αSMC-positive VSMCs in this region, a mechanism characteristic of AAA progression [22]. On the other hand, a slight increase in NRF2 was observed in the remaining contractile VSMCs in the intima and media, which was also clearly measurable in protein extracts from AAA tissue. However, further correlation analysis indicated that the increase in total NRF2 levels in tissue extracts was primarily attributed to its enhanced expression in immune cells rather than in VSMC.

In addition to the findings in aortic tissue, this study shows that the vulnerability to oxidative stress and the antioxidative stress response differ between aortic smooth muscle cells of different origins. In cells derived from AAA (AAA-SMC), the baseline level of NRF2 was significantly higher than in aortic VSMC derived from healthy donors. In line with previous findings, [10] this was observed in both aortic tissues and cell cultures. In contrast, NRF2 activity was higher in some but not all healthy VSMC than in AAA-SMC, indicating that there is heterogeneity even within apparently healthy cells. As no difference in the baseline level of the *NFR2* transcript (*NFE2L2*) was observed in untreated cells, we conclude that there are no internal disruptions in signalling pathways immediately upstream of *NRF2* transcription in AAA-SMC. At the same time, AAA-SMC were more vulnerable toward oxidative stress induced by H_2_O_2_ than healthy VSMC, but more robust against high dosages of the antioxidant reagent MitoQ. In AAA-SMC, long-term/low-dose MitoQ treatment significantly increased NRF2 activity, resulting in reduced ROS production during the 24 h recovery period following short-term H_2_O_2_ exposure, whereas no measurable effects were observed in healthy cells. Taken together, this suggests that MitoQ’s action as an electron transporter in mitochondria is particularly effective in AAA-SMC, where a large proportion of the mitochondria are defective [23,24,25]. In contrast, the immortalized VSMC cell line (iHAoSMC), used as a model for studies in this cell type, here displayed a high baseline level of NRF2 expression, but very low NRF2 activity, together with a comparatively higher sensitivity toward H_2_O_2_-induced oxidative stress and an increase in NRF2 activity in response to long-term/low-dose MitoQ treatment without resulting in reduction of ROS production.

MitoQ’s potential to reduce oxidative stress has been previously analysed in human keratinocytes, VSMC, and mouse endothelial cells, consistently demonstrating that its underlying mechanism of action is NRF2-dependent [15,16,26]. In addition, MitoQ has been shown to protect against oxidative stress-induced mitochondrial dysregulation in human cardiomyocytes [17]. Our study is in line with these previous investigations, demonstrating induction of NRF2 activity by MitoQ in AAA-SMC and in iHAoSMC. However, no significant NRF2-dependent response in healthy VSMC was observed under the subtoxic conditions used in this study. Moreover, ROS reduction in response to low-dose/long-term MitoQ treatment was only observed in AAA-SMC, but not in healthy cells or in iHAoSMC. This emphasizes the need to carefully analyse molecular effects of MitoQ treatment, with respect to the disease state of the cells, the MitoQ dosage, and the treatment time. The extent and exact molecular mechanism of MitoQ’s action may depend not only on the cell type but, in particular, on the activity of various signalling pathways currently present in the cells.

We and others observed that, unlike its parent ubiquinone coenzyme Q10, MitoQ might also have cytotoxic effects when used in high doses. Because of this property, MitoQ has also been tested as an anticancer agent. It was found to be 30-fold more cytotoxic to breast cancer cells than to healthy mammary cells by inducing a G1/S cell cycle arrest and checkpoint kinase activation in cancer cells without translating into caspase-dependent apoptotic events [27]. Here, we also found differences in the vulnerability of VSMC, depending on their disease state. At high doses (above 1 µM), MitoQ was 10 times more cytotoxic to healthy cells than to smooth muscle cells derived from AAA by inducing growth arrest without inducing apoptotic caspase 3/7. This indicates that AAA-SMC can tolerate higher MitoQ dosages than healthy VSMC. The reason for this difference is unclear so far. In breast cancer cells, MitoQ was accompanied by rapid mitochondrial membrane depolarization and cytochrome c release that may be partly responsible for the cytotoxic effect. Moreover, autophagy was induced by MitoQ at growth inhibitory concentrations in breast cancer cells, and an increase of superoxide generation and ROS production was observed [27]. Finally, the authors demonstrated that MitoQ induces oxidative modifications in the NRF2-inhibitor KEAP1, resulting in release and accumulation of NRF2 in the cytoplasm. Whether any of these mechanisms also accounts for the underlying effect of low-dose/long-term MitoQ treatment in VSMC is currently the subject of our further investigation. The fact that gene expression of NRF2 (NFEL2L) was increased in MitoQ-pretreated AAA-SMCs as a result of acute oxidative stress induced by H_2_O_2_ here suggests that MitoQ also acts on targets in signalling pathways upstream of NRF2 transcription.

VSMC located in a healthy aorta are considered to be non-proliferating and contractile, in contrast to the synthetic state in AAA, where the cells transdifferentiate into various specialized VSMC phenotypes and, at a later stage, undergo apoptosis and cell death [1,2,22]. Interestingly, a recent single-cell RNA sequencing analysis revealed that NRF2 regulates VSMC phenotypic switching in AAA [10]. In a murine model, the absence of NRF2 in VSMC exacerbated AAA formation, and downregulation of NRF2 promoted VSMC phenotype switching, elevating maximal aortic diameter, expression of inflammatory markers, and degradation of elastic fibers. In this context, our observation that MitoQ can increase NRF2 activity particularly in AAA-SMC without affecting healthy cells is promising and suggests that MitoQ may be effective as an adjunct in AAA therapy by reducing the phenotype switching of VSMCs, when applied at nontoxic concentrations. More importantly, our data demonstrate that long-term/low-dose MitoQ treatment reduced ROS production even under oxidative stress induction, particularly in AAA-SMC, which might help to reduce aortic wall remodelling and subsequent rupture risk.

Our findings are subject to a number of limitations that should be noted here. First, the number of AAA-SMC and healthy VSMC cultures used for our study was small, and the antioxidative stress response was also heterogenous in individual cell lines from both AAA patients and healthy donors. The reason for the latter is unclear so far. It is possible that different VSMC phenotypes were preferentially amplified from the tissue during the isolation process. Second, healthy VSMC derived from abdominal aorta are unfortunately not available for comparison. Instead, all commercially available healthy VSMC cultures are derived from thoracic aorta and therefore originate from a different embryonic source [28]. The impact of this different embryonic origin on the cells in the adult aorta is a matter of debate. While some differences appear to persist, others converge in the adult vessel [1,29]. Thus, we cannot rule out the possibility that the differing responses to oxidative stress and MitoQ between healthy VSMC and AAA-SMC are already determined by differences in embryonic origin. Alternatively, underlying causes for the heterogenic responses within the groups of healthy VSMC or AAA-SMC could include innate, patient-specific differences in NRF2 activation. Although our data do not allow us to definitively answer this question, a significant difference in oxidative stress response was consistently observed, here between AAA-SMCs and healthy VSMCs. Considering our results obtained in the immortalized cell line iHAoSMC, one should be aware that these cells behave only partially like their parental primary cells. Third, we used hydrogen peroxide as an inducer of oxidative stress. Although H_2_O_2_ is the most common and abundant ROS in cells, [30] it is known to be unstable in cell culture supernatants and can freely pass through the cell membrane and be released. Thus, its concentration interferes with endogenous metabolism of the cell during the experiments. In order to minimize uncontrollable fluctuations in H_2_O_2_ concentration, only the response to an acute, 30-min challenge following prolonged pre-treatment with MitoQ was tested here, followed by recovery periods of varying lengths. We are aware that this can only model the complex environment in the AAA, with its inflammatory cells and mechanical stress, to a limited extent.

Despite the limitations of studies in cell cultures, some of our findings were in line with our observations in aortic tissues derived from patients undergoing surgery and consistent with the findings by others [10]. Despite a clear loss of VSMC in the media and intima of AAA-derived aortas, our data indicate a significantly increased amount of NRF2. In addition, the observed increase in NRF2 target gene expression suggests a higher NRF2 activity in AAA tissues. At first glance, this sounds like a contradiction. Why cannot the increased NRF2 activity in VSMCs prevent or slow the progression of AAA, given that it has an antioxidant effect and prevents phenotype switching in VSMCs in the aorta? The answer to this question likely lies in the dynamics of the system. Once a certain threshold is reached, NRF2 can obviously no longer compensate for the increased ROS production, leading to inflammation, senescence, and, finally, loss of VSMCs, which in turn progressively weakens the aortic wall. Pharmaceuticals that support activation of the NRF2 system and protect mitochondria by reducing ROS production are therefore particularly promising for the treatment of AAA patients. It should be noted, however, that an increase of NRF2 by these pharmaceuticals will only be effective if NFR2 is also active as a transcription factor and can initiate transcription of antioxidative target genes. Binding to the ARE, as it is observed by commercially available NRF2 activity tests, is not sufficient. We cannot conclude from our data whether the binding to the DNA target sequence results in full NRF2-induced gene transcription because only part of the tested NRF2 target genes displayed increased gene expression upon treatment of the cells with MitoQ.

Several recent animal studies convincingly confirmed the importance of NRF2 as a potential pharmaceutical target for AAA reduction. The compounds sulforaphane [31] and berberine [32] were shown to prevent AAA formation through the inhibition of phenotypic switching of VSMC via the activation of NRF2. In addition, compounds such as itaconate [33] and cardamonin [34] were shown to reduce AAA formation via acting on the NRF2 signalling pathway. Based on our cell culture data, we cannot yet conclude whether the MitoQ-induced activation of NRF2 will have a similar protective effect on AAA in vivo. However, taking into account previous clinical trials on the improvement of vascular function through treatment with MitoQ, [35,36,37] supplementation in patients with small AAA also appears promising. In summary, our study adds important findings on the heterogenous response to pharmaceutical treatment of VSMC derived from different sources and supports the idea to use antioxidative, NRF2-targenting reagents, such as MitoQ, for prevention of AAA progression in patients.

## 5. Conclusions

Our data show that even within the same cell type (aortic VSMC), the NRF2-mediated cellular responses to oxidative stress and to treatment with the antioxidant MitoQ differ significantly from one another. It is thus possible that within the wall of an aortic aneurysm, the NRF2 signalling pathway is still active in some VSMC subtypes while it is impaired in others. Since, according to our study and others, MitoQ exerts part of its efficacy by activating NRF2, it might therefore only be partially effective in the aneurysm wall. However, the exact mechanism of action of MitoQ in relation to its interaction with mitochondria in aortic VSMC requires further investigation before a clinical trial in patients with small AAA may be considered.

## Data Availability

All processed data used to interpret the results are contained in the article or Supplementary Materials. The raw data supporting the conclusions of this study are available upon reasonable request from the authors. Part of the data is not publicly available in order to protect the privacy of the participants involved.

## Supplementary Materials

The following supporting information can be downloaded at: https://www.mdpi.com/article/10.3390/cells15161499/s1.

## Abbreviations

The following abbreviations are used in this manuscript:

8-OHDG	8-hydroxyguanosine
αSMA	alpha smooth muscle actin
AAA	abdominal aortic aneurysm
ACTB	beta-Actin gene
ARE	antioxidative response element
CoQ10	coenzyme Q10
CD3	cluster of differentiation 3
CD45	cluster of differentiation 45
CD62L	cluster of differentiation 62L
DAB	diaminobenzidine
ECL	enhanced chemiluminescence
ECM	extracellular matrix
FFPE	formalin-fixed and paraffin-embedded
GAPDH	glycerinaldehyde-3-phosphat-dehydrogenase
HBV	hepatitis B virus
HCV	hepatitis C virus
HIV	human immunodeficiency virus
HO-1	heme oxygenase 1
HRP	horse reddish peroxidase NADPH
IC50	half maximal inhibitory concentration
iHAoSMC	immortalized human aortic smooth muscle cells
KEAP1	Kelch-like-ECH-associated protein 1
NADPH	nicotinamide adenine dinucleotide phosphate
NOX	NADPH oxidaseNRF2/
NFE2L2	nuclear factor erythroid 2-related factor 2
ROS	reactive oxygen species
SMC-GM2	Smooth Muscle Cell Growth Medium 2
SOD2	superoxide dismutase 2/manganese superoxide dismutase (MnSOD)
TBST	tris-buffered saline with Tween 20
TXNRD1	thioredoxin reductase 1
(V)SMCs	(vascular) smooth muscle cells
VBBH	Vascular Biobank Heidelberg

## References

[1] Hu Y., Cai Z., He B. (2023). Smooth Muscle Heterogeneity and Plasticity in Health and Aortic Aneurysmal Disease. Int. J. Mol. Sci..

[2] Lu H., Du W., Ren L., Hamblin M.H., Becker R.C., Chen Y.E., Fan Y. (2021). Vascular Smooth Muscle Cells in Aortic Aneurysm: From Genetics to Mechanisms. J. Am. Heart Assoc..

[3] Bennett M.R., Sinha S., Owens G.K. (2016). Vascular Smooth Muscle Cells in Atherosclerosis. Circ. Res..

[4] Basatemur G.L., Jorgensen H.F., Clarke M.C.H., Bennett M.R., Mallat Z. (2019). Vascular smooth muscle cells in atherosclerosis. Nat. Rev. Cardiol..

[5] Rombouts K.B., van Merrienboer T.A.R., Ket J.C.F., Bogunovic N., van der Velden J., Yeung K.K. (2022). The role of vascular smooth muscle cells in the development of aortic aneurysms and dissections. Eur. J. Clin. Investig..

[6] Petsophonsakul P., Furmanik M., Forsythe R., Dweck M., Schurink G.W., Natour E., Reutelingsperger C., Jacobs M., Mees B., Schurgers L. (2019). Role of Vascular Smooth Muscle Cell Phenotypic Switching and Calcification in Aortic Aneurysm Formation. Arter. Thromb. Vasc. Biol..

[7] Reddy V.P. (2023). Oxidative Stress in Health and Disease. Biomedicines.

[8] Badran A., Nasser S.A., Mesmar J., El-Yazbi A.F., Bitto A., Fardoun M.M., Baydoun E., Eid A.H. (2020). Reactive Oxygen Species: Modulators of Phenotypic Switch of Vascular Smooth Muscle Cells. Int. J. Mol. Sci..

[9] He F., Ru X., Wen T. (2020). NRF2, a Transcription Factor for Stress Response and Beyond. Int. J. Mol. Sci..

[10] Xiao X., Li C., Huang X., Chen G., Huang X., Song F., Zhou Y., Liu X., Zhou X., Meng J. (2024). Single-cell RNA sequencing reveals that NRF2 regulates vascular smooth muscle cell phenotypic switching in abdominal aortic aneurysm. FASEB J..

[11] Ross M.F., Prime T.A., Abakumova I., James A.M., Porteous C.M., Smith R.A., Murphy M.P. (2008). Rapid and extensive uptake and activation of hydrophobic triphenylphosphonium cations within cells. Biochem. J..

[12] Pang M., Wang S., Shi T., Chen J. (2025). Overview of MitoQ on prevention and management of cardiometabolic diseases: A scoping review. Front. Cardiovasc. Med..

[13] Gioscia-Ryan R.A., Battson M.L., Cuevas L.M., Eng J.S., Murphy M.P., Seals D.R. (2018). Mitochondria-targeted antioxidant therapy with MitoQ ameliorates aortic stiffening in old mice. J. Appl. Physiol..

[14] Ning R., Li Y., Du Z., Li T., Sun Q., Lin L., Xu Q., Duan J., Sun Z. (2021). The mitochondria-targeted antioxidant MitoQ attenuated PM_2.5_-induced vascular fibrosis via regulating mitophagy. Redox Biol..

[15] Cui L., Zhou Q., Zheng X., Sun B., Zhao S. (2020). Mitoquinone attenuates vascular calcification by suppressing oxidative stress and reducing apoptosis of vascular smooth muscle cells via the Keap1/Nrf2 pathway. Free Radic. Biol. Med..

[16] Cen M., Ouyang W., Zhang W., Yang L., Lin X., Dai M., Hu H., Tang H., Liu H., Xia J. (2021). MitoQ protects against hyperpermeability of endothelium barrier in acute lung injury via a Nrf2-dependent mechanism. Redox Biol..

[17] Parker A.M., Lees J.G., Tate M., Phang R.J., Velagic A., Deo M., Bishop T., Krieg T., Murphy M.P., Lim S.Y. (2025). MitoQ Protects Against Oxidative Stress-Induced Mitochondrial Dysregulation in Human Cardiomyocytes. J. Mol. Cell. Cardiol. Plus.

[18] Erhart P., Cakmak S., Grond-Ginsbach C., Hakimi M., Bockler D., Dihlmann S. (2019). Inflammasome activity in leucocytes decreases with abdominal aortic aneurysm progression. Int. J. Mol. Med..

[19] Dihlmann S., Erhart P., Mehrabi A., Nickkholgh A., Lasitschka F., Bockler D., Hakimi M. (2014). Increased expression and activation of absent in melanoma 2 inflammasome components in lymphocytic infiltrates of abdominal aortic aneurysms. Mol. Med..

[20] Livak K.J., Schmittgen T.D. (2001). Analysis of relative gene expression data using real-time quantitative PCR and the 2(-Delta Delta C(T)) Method. Methods.

[21] Wortmann M., Skorubskaya E., Peters A.S., Hakimi M., Bockler D., Dihlmann S. (2019). Necrotic cell debris induces a NF-kappaB-driven inflammasome response in vascular smooth muscle cells derived from abdominal aortic aneurysms (AAA-SMC). Biochem. Biophys. Res. Commun..

[22] Qian G., Adeyanju O., Olajuyin A., Guo X. (2022). Abdominal Aortic Aneurysm Formation with a Focus on Vascular Smooth Muscle Cells. Life.

[23] Tavris B.S., Peters A.S., Bockler D., Dihlmann S. (2023). Mitochondrial Dysfunction and Increased DNA Damage in Vascular Smooth Muscle Cells of Abdominal Aortic Aneurysm (AAA-SMC). Oxid. Med. Cell. Longev..

[24] Liu Y., Yu M., Wang H., Dorsey K.H., Cheng Y., Zhao Y., Luo Y., Zhao G., Zhao Y., Lu H. (2025). Restoring Vascular Smooth Muscle Cell Mitochondrial Function Attenuates Abdominal Aortic Aneurysm in Mice. Arter. Thromb. Vasc. Biol..

[25] Ouyang M., Wang M., Yu B. (2021). Aberrant Mitochondrial Dynamics: An Emerging Pathogenic Driver of Abdominal Aortic Aneurysm. Cardiovasc. Ther..

[26] Zhao Y., Xiong R., Jin S., Li Y., Dong T., Wang W., Song X., Guan C. (2025). MitoQ alleviates H_2_O_2_-induced mitochondrial dysfunction in keratinocytes through the Nrf2/PINK1 pathway. Biochem. Pharmacol..

[27] Rao V.A., Klein S.R., Bonar S.J., Zielonka J., Mizuno N., Dickey J.S., Keller P.W., Joseph J., Kalyanaraman B., Shacter E. (2010). The antioxidant transcription factor Nrf2 negatively regulates autophagy and growth arrest induced by the anticancer redox agent mitoquinone. J. Biol. Chem..

[28] Majesky M.W. (2007). Developmental basis of vascular smooth muscle diversity. Arter. Thromb. Vasc. Biol..

[29] Pfaltzgraff E.R., Shelton E.L., Galindo C.L., Nelms B.L., Hooper C.W., Poole S.D., Labosky P.A., Bader D.M., Reese J. (2014). Embryonic domains of the aorta derived from diverse origins exhibit distinct properties that converge into a common phenotype in the adult. J. Mol. Cell. Cardiol..

[30] de Almeida A., de Oliveira J., da Silva Pontes L.V., de Souza Junior J.F., Goncalves T.A.F., Dantas S.H., de Almeida Feitosa M.S., Silva A.O., de Medeiros I.A. (2022). ROS: Basic Concepts, Sources, Cellular Signaling, and its Implications in Aging Pathways. Oxid. Med. Cell. Longev..

[31] Huang Z., Tao Y., Liu C., Wang J., Deng C., Han Z., Yun T., Li T., Lu W., Lai Y. (2026). Sulforaphane prevents abdominal aortic aneurysm formation through the inhibition of phenotypic switching of vascular smooth muscle cells and inflammation via the activation of Nrf2. Cell. Signal..

[32] Li S., Xiao X., Chang Y., Xu Z., Zheng X., Zhou H., Ding H., Lu W., Li T., Tao Y. (2025). Berberine Inhibits Abdominal Aortic Aneurysm Formation and Vascular Smooth Muscle Cell Phenotypic Switching by Regulating the Nrf2 Pathway. J. Cell. Mol. Med..

[33] Song H., Xu T., Feng X., Lai Y., Yang Y., Zheng H., He X., Wei G., Liao W., Liao Y. (2020). Itaconate prevents abdominal aortic aneurysm formation through inhibiting inflammation via activation of Nrf2. EBioMedicine.

[34] Tsai H.Y., Hsu Y.J., Lin C.Y., Huang P.H., Hsu C.W., Tsai S.H. (2025). Cardamonin attenuates angiotensin II-induced abdominal aortic aneurysms through activation of the Nrf2/HO-1 pathway. Int. J. Cardiol. Heart Vasc..

[35] Rossman M.J., Gioscia-Ryan R.A., Clayton Z.S., Murphy M.P., Seals D.R. (2020). Targeting mitochondrial fitness as a strategy for healthy vascular aging. Clin. Sci..

[36] Rossman M.J., Santos-Parker J.R., Steward C.A.C., Bispham N.Z., Cuevas L.M., Rosenberg H.L., Woodward K.A., Chonchol M., Gioscia-Ryan R.A., Murphy M.P. (2018). Chronic Supplementation with a Mitochondrial Antioxidant (MitoQ) Improves Vascular Function in Healthy Older Adults. Hypertension.

[37] Carlini N.A., Harber M.P., Fleenor B.S. (2024). Acute effects of MitoQ on vascular endothelial function are influenced by cardiorespiratory fitness and baseline FMD in middle-aged and older adults. J. Physiol..

