# Supplementary material for: MitoQ Has Diverse Effects on H2O2-Induced Oxidative Stress and the NRF2 Signalling Pathway in Aortic Smooth Muscle Cells of Different Origins
Source: Cells. 2026 Aug 20;15(16):1499. doi: 10.3390/cells15161499 (PMC13510859; doi:10.3390/cells15161499)
Supplement: Supplementary file 1 [file cells-15-01499-s001.zip › Supplementary tables Haas et al_2026_anonym.pdf]

Supplementary table S1: Primer sequences

Gene name	Forward primer (5’ -> 3’)	Reverse primer (5’ -> 3’)
NLF2L2 (NRF2)	CGCAGACATTCCCGTTTGTA	AGCAATGAAGACTGGGCTCT
HMOX1 (HO-1)	CGGGCCAGCAACAAAGTG	GAGTGTAAGGACCCATCGGA
TXNRD1	TGGAATCCACCCTGTCTGTG	TGGTCACAGATGGGGCTTAA
GPX2	GGCTTCCCTTGCAACCAATT	TTGGACAAGGGTGAAGGTGG
SOD2	AAGGAACGGGGACACTTACA	AGCAGTGGAATAAGGCCTGT
CD62/SELL	GGAATCTGGTCAAATCCTAGTCC	TGCCACTGGAATGAAGAGGG
CD3	GGAGTCTTCTGCTTTGCTGG	GATCTCGGAGGGGCTGATAG
ACTA2 (αSMA)	CCAGCACCATGAAGATCAAG	ACATCTGCTGGAAGGTGGAC
MYH11	TGAACGCACTCAAGAGCAAG	TCCGTTTCCTCCTCAGAACC
TAGLN	TTCAAGCAGATGGAGCAGGT	CTGCCATGTCTTTGCCTTCA
GAPDH	GGCTGCTTTTAACTCTGGTA	CTTGACGGTGCCATGGAATT
ACTB	CACCATGTACCCTGGCATTG	AGTACTTGCGCTCAGGAGG

Supplementary table S2: Characteristics of primary HAoSMC cultures

ID	origin	Provider	Age of the patient	sex
**HAoSMC-424Z011.4**	Aorta, thoracic	PromoCell, Heidelberg, Germany	14	male
**HAoSMC-485Z024.2**	Aorta, thoracic	PromoCell, Heidelberg, Germany	72	male
**HAoSMC-505Z028.1**	Aorta, thoracic	PromoCell, Heidelberg, Germany	50	male
**HAoSMC-505Z009.1**	Aorta, thoracic	PromoCell, Heidelberg, Germany	82	female
**AAA-SMC-1**	Abdominal aortic aneurysm	VBBH, Germany	59	male
**AAA-SMC-2**	Abdominal aortic aneurysm	VBBH, Germany	57	female
**AAA-SMC-3**	Abdominal aortic aneurysm	VBBH, Germany	46	male
**AAA-SMC-4**	Abdominal aortic aneurysm	VBBH, Germany	73	male
