# Supplementary material for: MitoQ Has Diverse Effects on H2O2-Induced Oxidative Stress and the NRF2 Signalling Pathway in Aortic Smooth Muscle Cells of Different Origins
Source: Cells. 2026 Aug 20;15(16):1499. doi: 10.3390/cells15161499 (PMC13510859; doi:10.3390/cells15161499)
Supplement: Supplementary file 1 [file cells-15-01499-s001.zip › Supplemetal figures S1-S6 Haas et al_2026_R1.pdf]

Supplemental figures to

MitoQ has diverse effects on H_2O_2-induced oxidative stress and the NRF2 signalling pathway in aortic smooth muscle cells of different origins.

By Simon Haas, Bingchen Hou, Andreas Peters, Johannes Hatzl, Dittmar Böckler and Susanne Dihlmann

Supplemental Figure S1

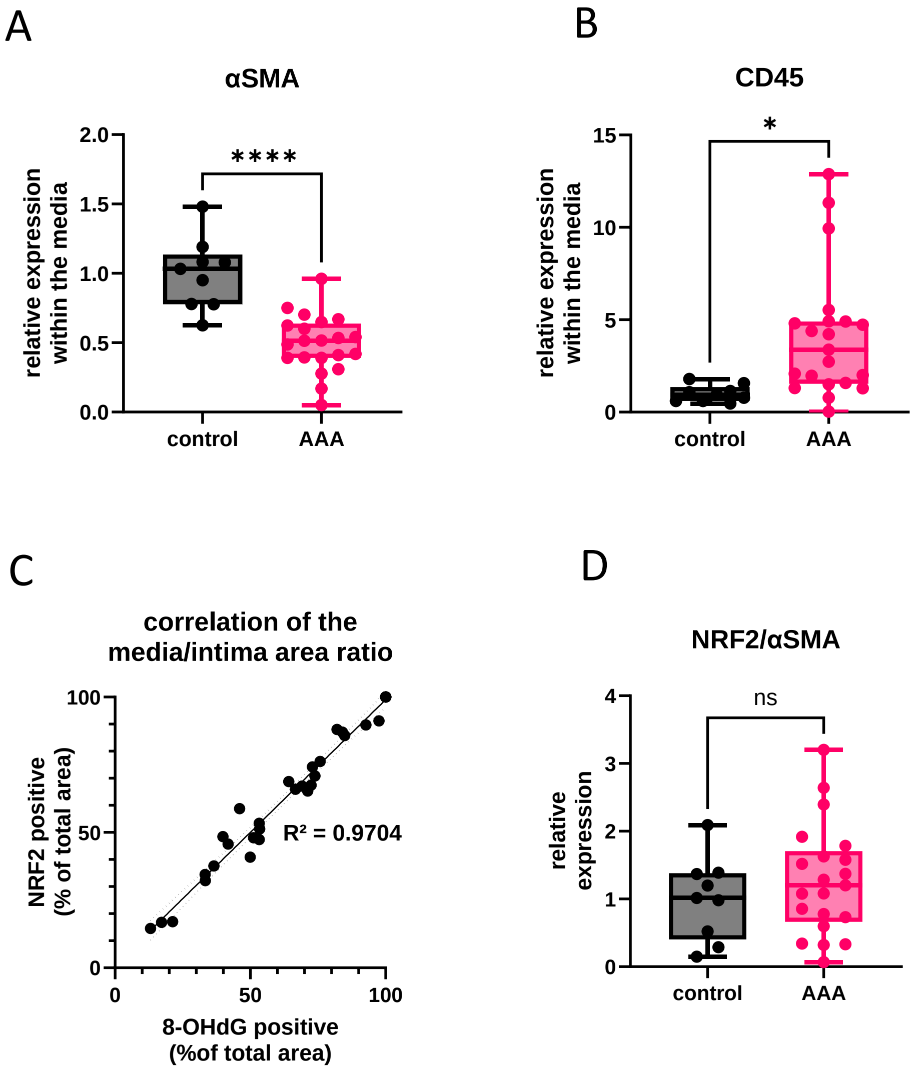

Supplemental figure S1: Quantification of the immunohistochemical analysis. (A) Relative expression of αSMA, representing the proportion of VSMC within the media/intima. (B) Relative expression of CD45, representing the proportion of immune cells within the media/intima. (C) correlation of media/intima area ratios positive for NRF2 versus 8-OHdG. (D) relative expression of NRF2 in VSMC (αSMC positive areas). Data were analysed by unpaired t test comparing n = 9 control and n = 21 AAA samples. *: $P < 0.05$, ****: $P < 0.0001$, n.s.: not significant.

Supplemental Figure S2

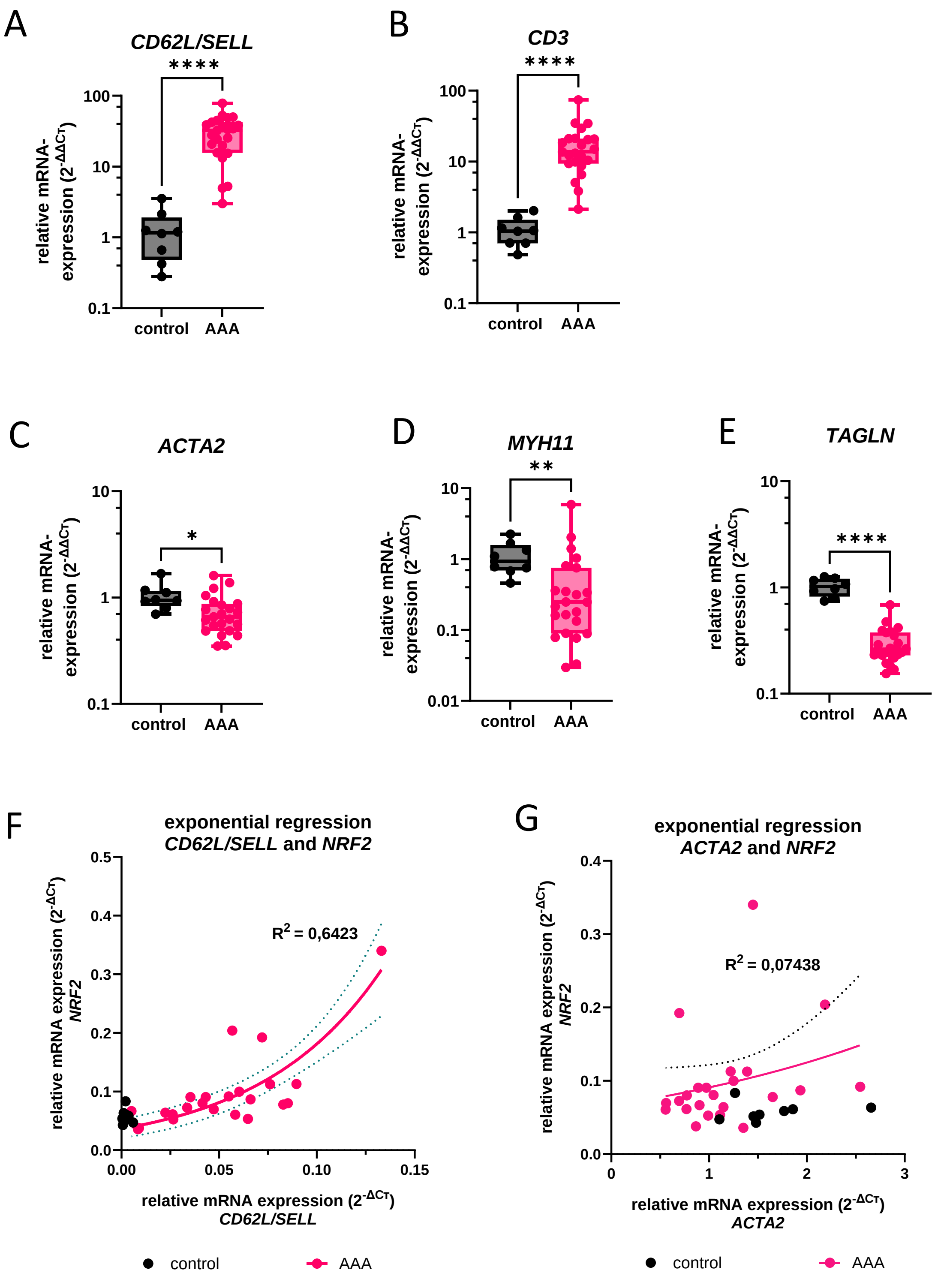

Supplemental figure S2: Relative transcript expression of markers for VSMC and immune cells in whole cell extracts derived from AAA and control tissues. (A) *CD62L/SELL*, (B) *CD3*, (C) *ACTA2*, (D) *MYH11*, (E) *TAGLN* transcript expression. Box plots show the median, maximum and minimum of $2^{-\Delta\Delta Ct}$ values derived from real-time qPCR. Data were analysed by Mann-Whitney test comparing n = 8 control and n = 23 AAA samples. *: P < 0.05, ****: P < 0.0001, n.s.: not significant. (F) and (G): Nonlinear regression analysis of the relative transcript expressions of *NRF2* and the imunne celll marker *CD62/SELL* (F) and expressions of *NRF2* and the VSMC marker *ACTA2* (G) in toal cell extracts derived from n = 8 control and n = 22 AAA samples.

Supplemental figure S3

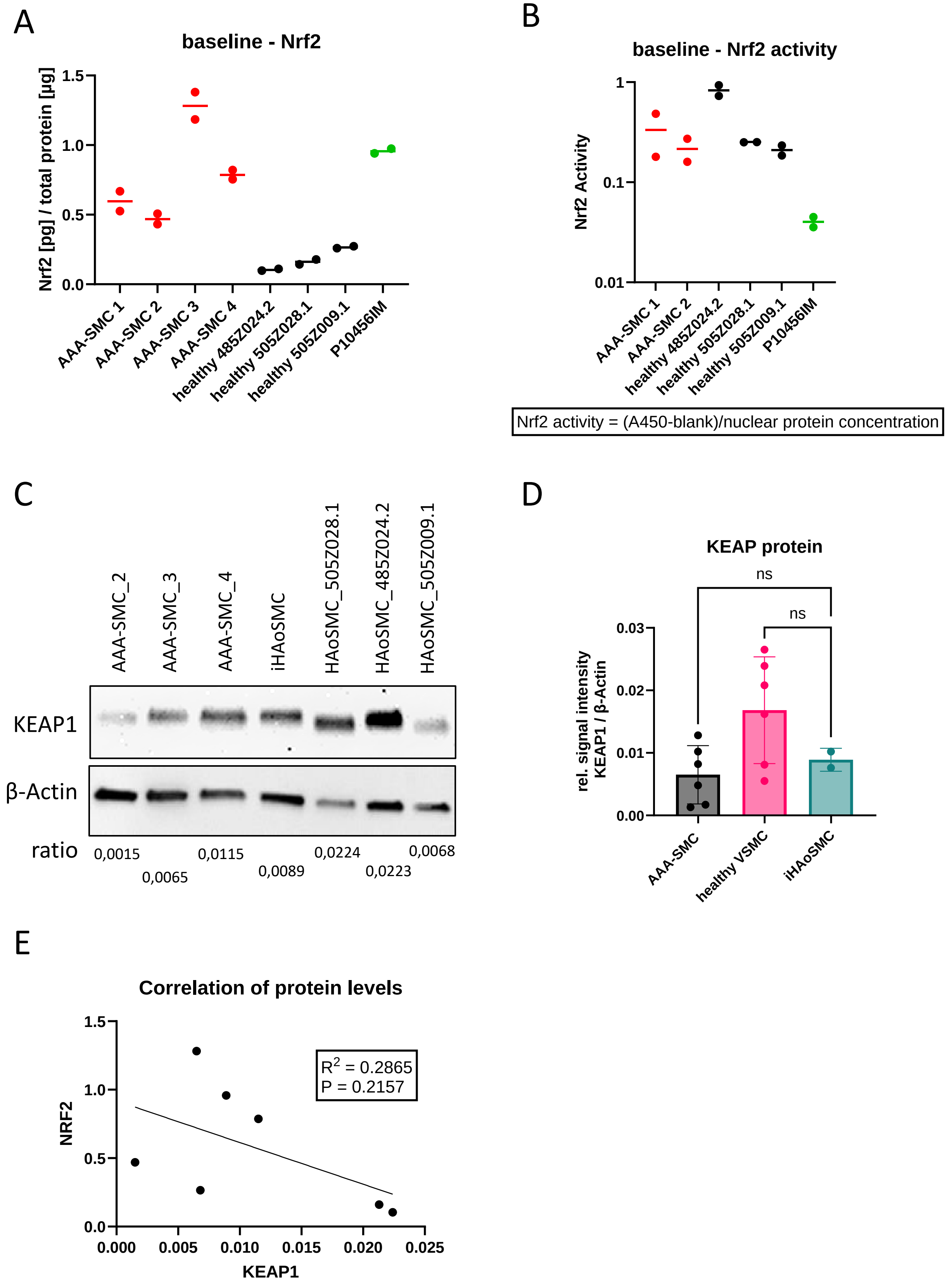

Supplemental figure S3. NFR2 and KEAP1 in individual cell cultures. (A) NRF2 protein levels were determined by ELISA and normalized to the total protein level of each sample. Data are shown as pg NRF2 per µg protein. (B) NRF2 binding activity to DNA was assayed from nuclear extracts by using an colorimetric NRF2 activity assay. Data were derived from n = two AAA-SMC (AAA), n = 3 healthy SMC (healthy) and one immortalized cell lines (IM). The median and two technical repeats are shown for each cell culture/cell line. (C) KEAP1 protein levels were determined by Western Blotting using KEAP1 (D6B12) Rabbit mAb (Cell signaling technology, # 8047) (D) Signal intensities were quantified by ImageJ/FIJI and normalized to β-Actin levels for semi-quantitative comparison. (E) Simple linear regression of mean KEAP1 vs NRF2 protein levels. Each dot corresponds to one of the cell lines. GraphPad Prism 10.6.1. was used for analysis of A, B, C and D. ns = not significant (ordinary one-way ANOVA).

Supplemental figure S4

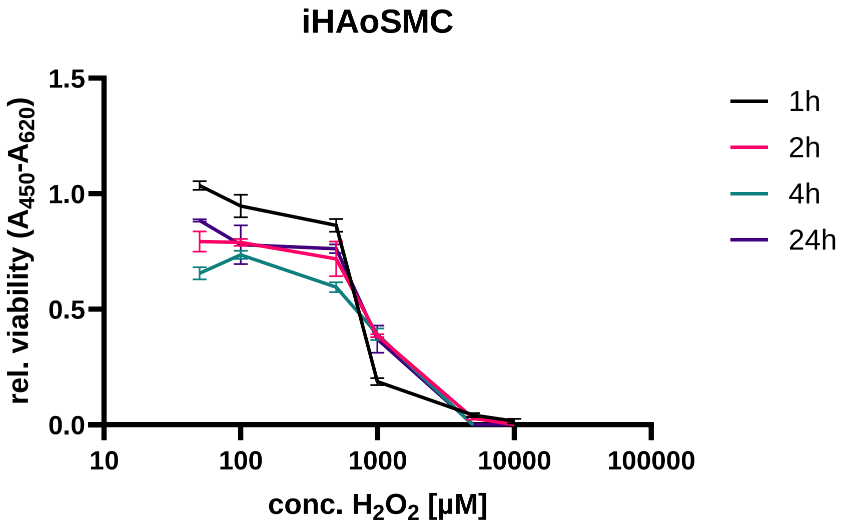

Supplemental figure S4: Dose response of iHAoSMC to chronic oxidative stress induced by H_2O_2. Cells were treated with increasing doses of H_2O_2 for 1, 2, 4, and 24 hours. Viability was evaluated by WST-1 proliferation reagent. Data represent the mean and SD of three measurements.

Supplemental figure S5

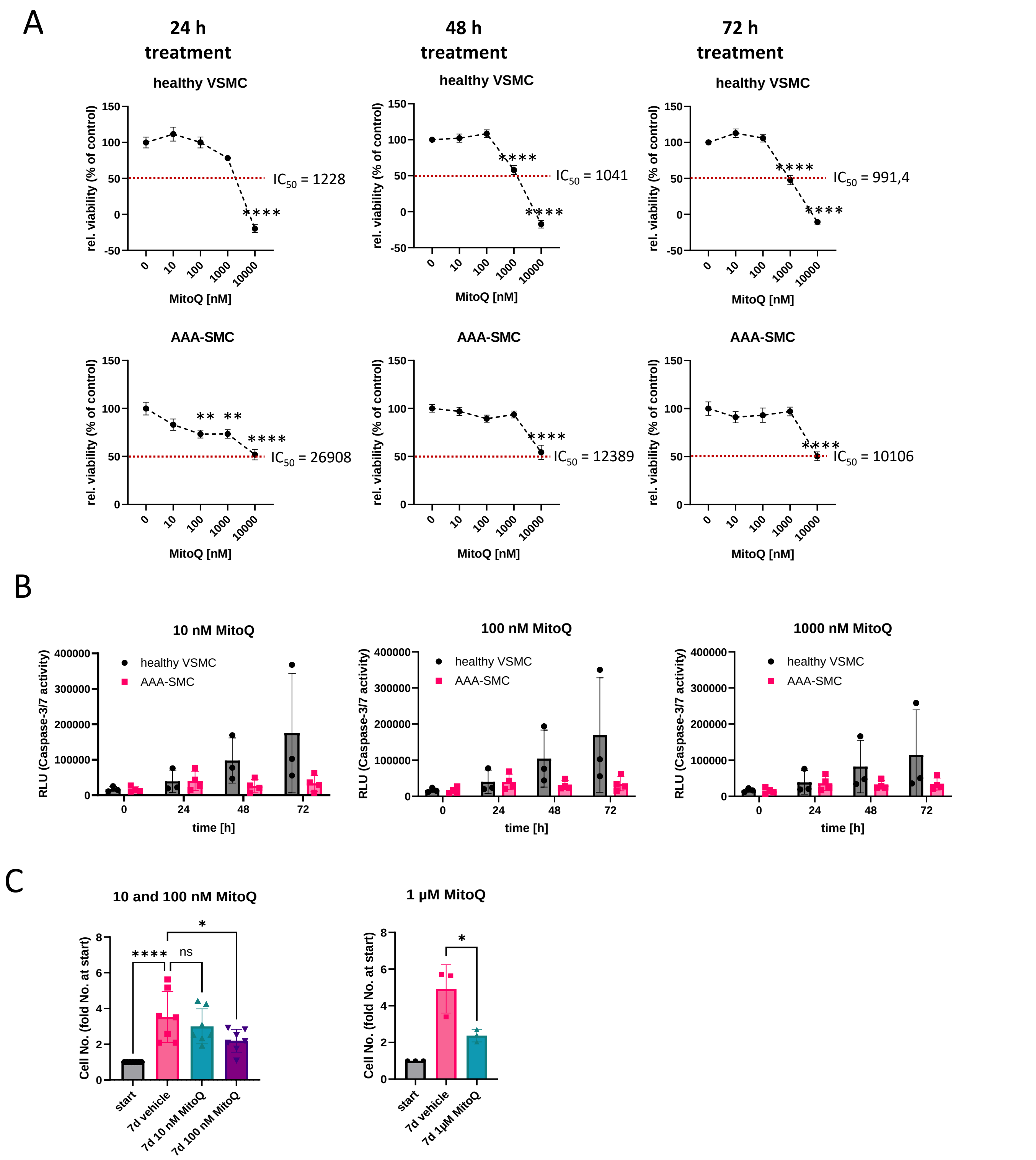

Supplemental Figure S5. MitoQ affects the viability and growth of healthy VSMC and AAA-SMC. **A.** Viability after 24h, 48h and 72 h 9991,with increasing concentrations of MitoQ. Shown are the means with SEM of n = 3 healthy VSMC or n = 3 AAA-SMC cultures, measured in triplicates using a WST-1 viability assay. The IC50 was calculated by non-linear regression using GraphPadPrism, ([inhibitor] vs normalized response – variable slope). **B.** Apoptosis after treatment with 10 nM, 100 nM and 1000 nM MitoQ for varying periods of times, as determined by a luminescent Caspase-Glo® 3/7 Assay (Promega). Shown are the means with SEM of n = 3 healthy or n = 3 aneurysmatic cell cultures, measured in triplicates. **C.** Relative cell numbers after 7d of treatment with various concentrations of MitoQ. Left panel: 2.4 x 10^5 cells of *n = 7* aortic SMC cultures were seeded in cell culture flasks and treated with two different concentrations of MitoQ (10 nM or 100 nM) or vehicle for seven days. Right panel: 5 x 10^5 cells of *n = 3* aortic SMC cultures were treated with MitoQ (1 µM) or vehicle for seven days. The cell count was determined manually under the microscope using a Neubauer counting chamber. Growth is shown as fold cell number compared with start (1.0). Individual response of the three healthy and five AAA-SMC are shown in the supplementary data. Statistical analysis: Ordinary one-way ANOVA with adjusted P-values (Dunnett's multiple comparison test). Ns: not significant, *: $P < 0.05$, **: $P < 0.01$, ****: $P < 0.0001$.

Supplemental figure S6

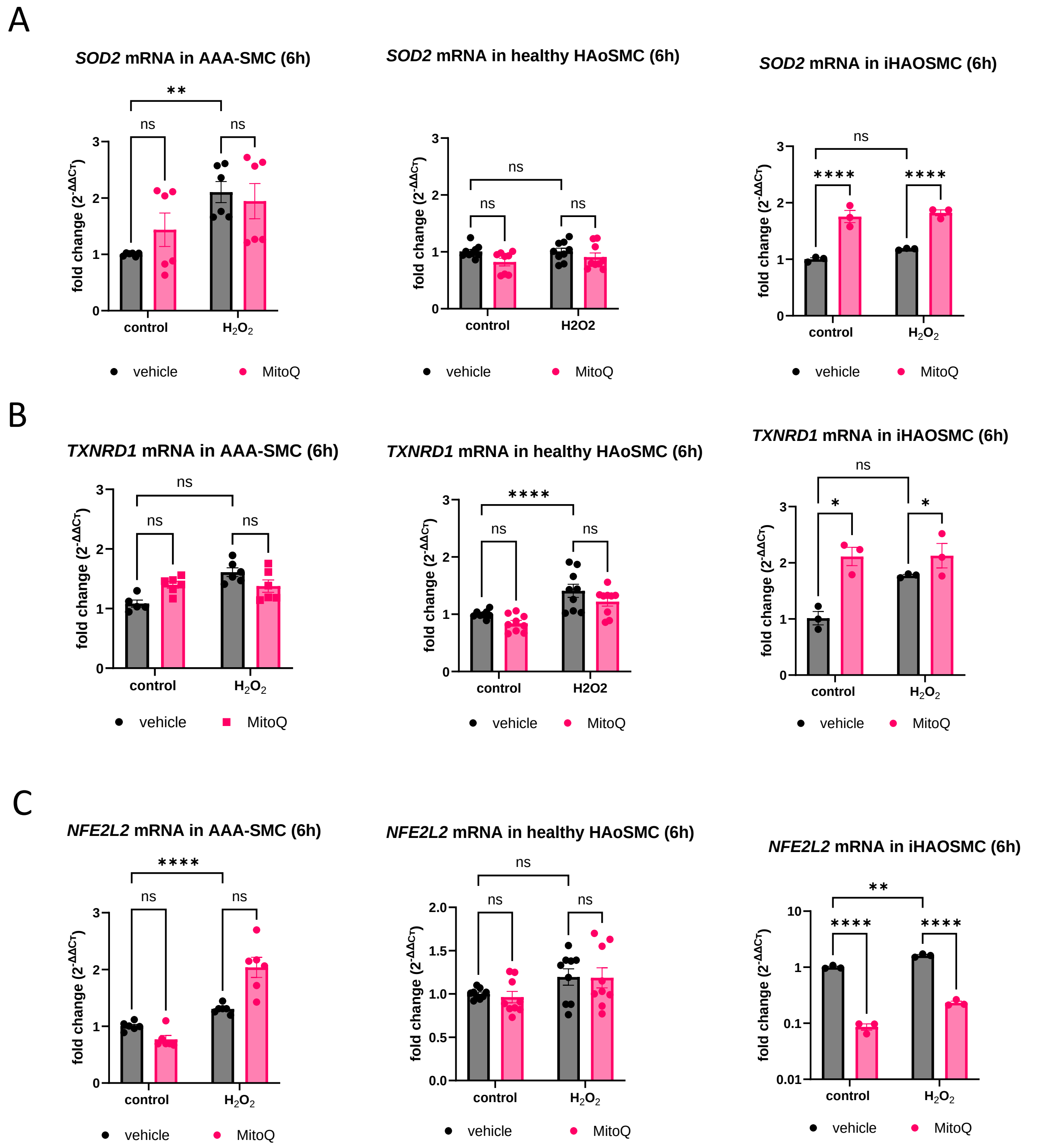

Supplemental Figure S6. Transcriptional expression, after 30 min. H_2O_2 treatment and 6h of recovery. (**A**) *SOD2* (**B**) *TXNRD1* ***(C)*** *NFE2L2 (NRF2)* transcript expression was analysed by qPCR using specific primers. Data show the 2^-(ΔCt) values. Data were derived from n = two AAA-SMC (AAA), n = 3 healthy SMC (healthy) and one immortalized cell line (IM). Bars represent the mean ± SEM. Data were statistically analysed by ordinary one-way ANOVA and Tukey's multiple comparison test; ns: not significant, *: $P < 0.05$; ** $P < 0.01$, ****: $P < 0.0001$.
