# Supplementary material for: MitoQ Has Diverse Effects on H2O2-Induced Oxidative Stress and the NRF2 Signalling Pathway in Aortic Smooth Muscle Cells of Different Origins
Source: Cells. 2026 Aug 20;15(16):1499. doi: 10.3390/cells15161499 (PMC13510859; doi:10.3390/cells15161499)
Supplement: Supplementary file 1 [file cells-15-01499-s001.zip › original images of Western blots.pdf]

Original images of Western Blots in Figure 2B

Please note: The images shown do not correspond to the original resolutions used for the analyses. Due to compression for display as a PDF file and the resulting reduction in pixel count, details may appear distorted.

Nuclear extracts

NRF2 ECL + Marker

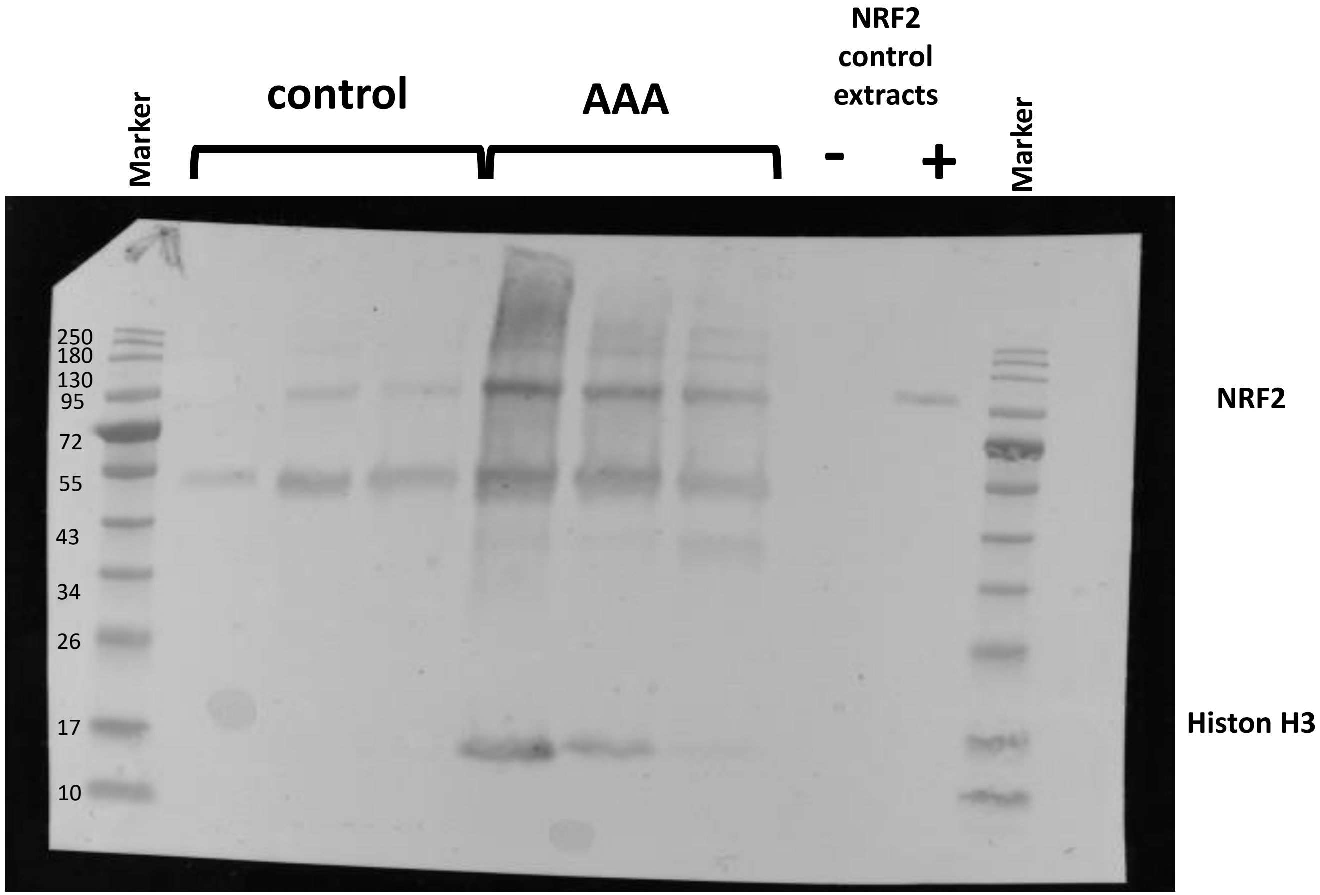

NRF2 ECL + Histon H3 ECL + Marker

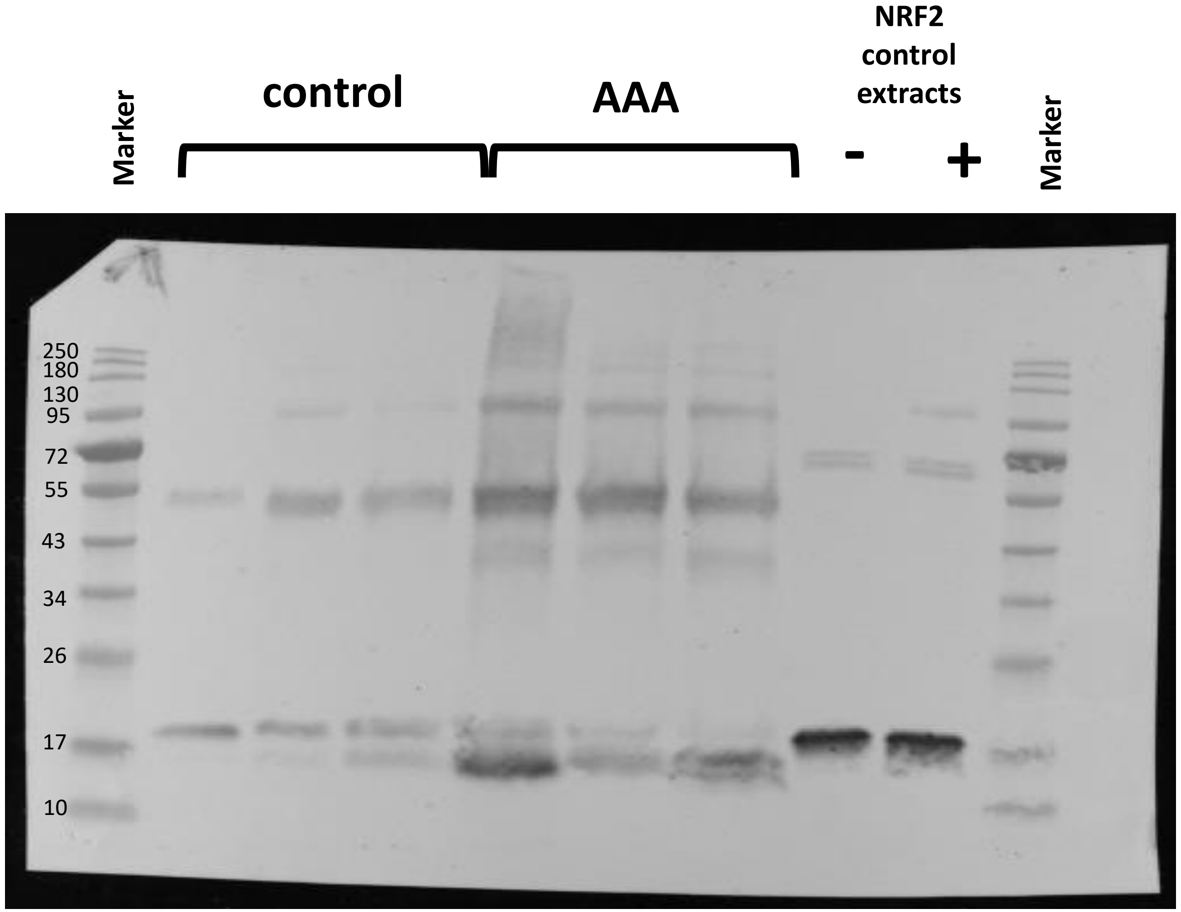

Nuclear extracts

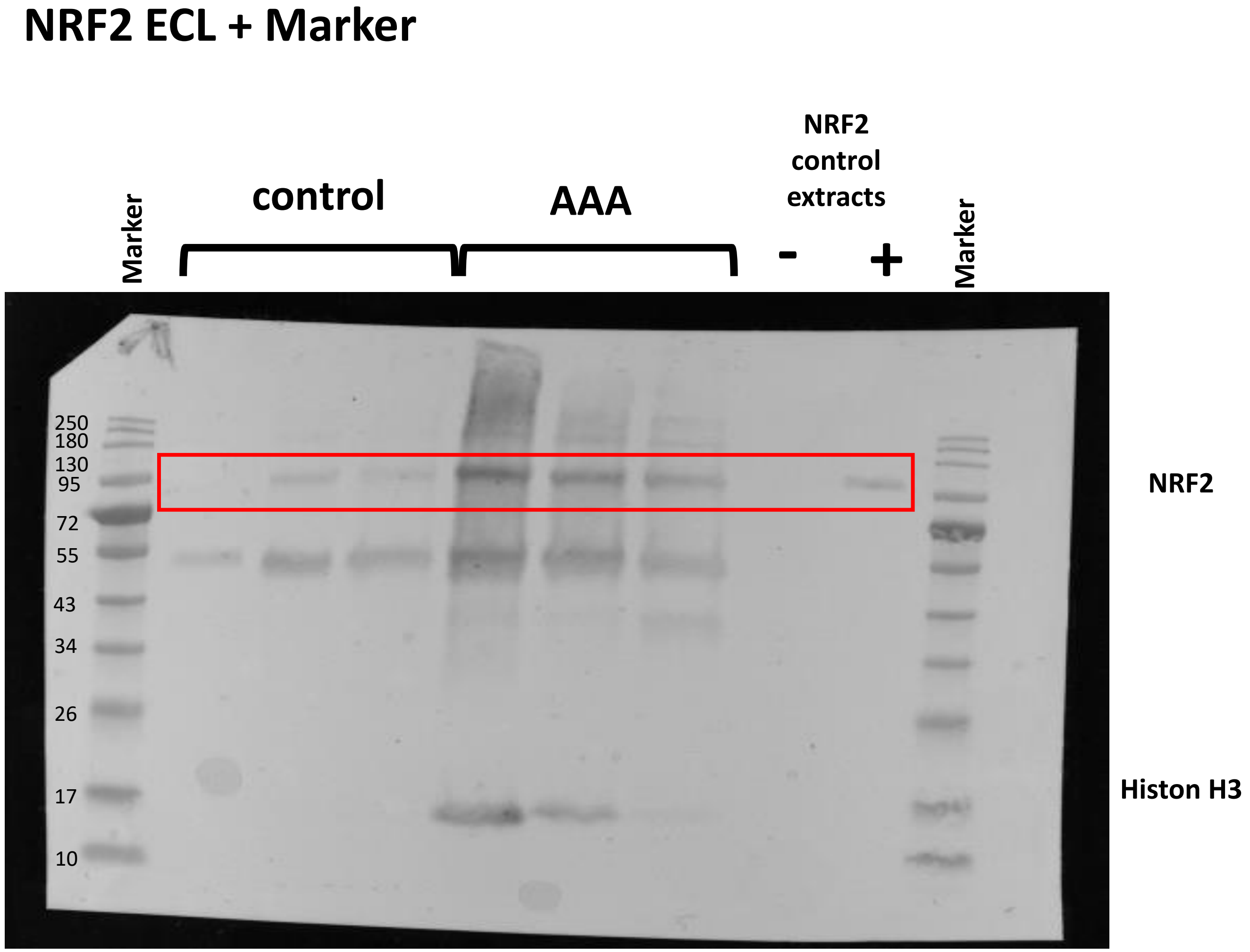

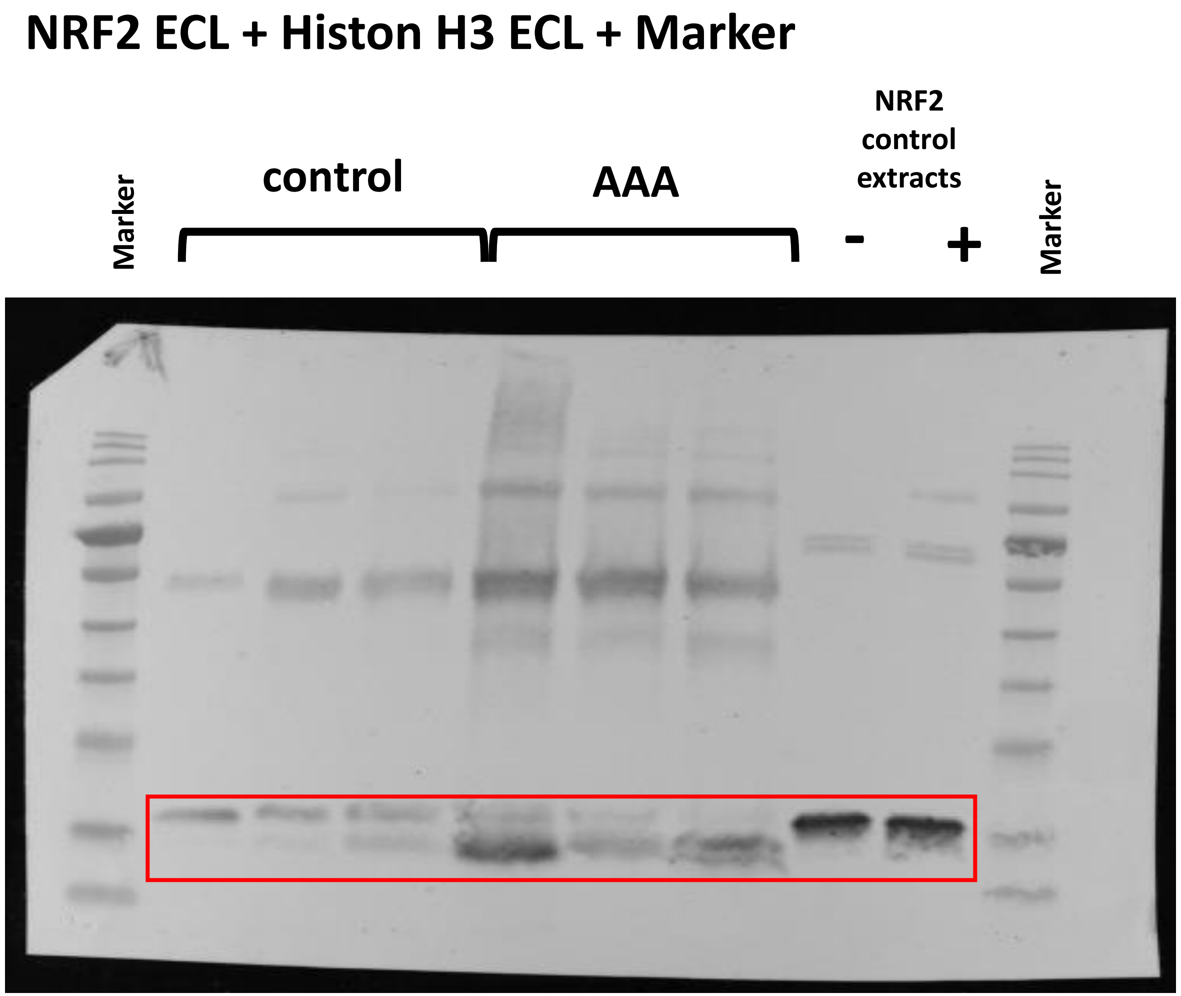

Area used for the figure

Cytoplasm

NRF2 ECL + Marker

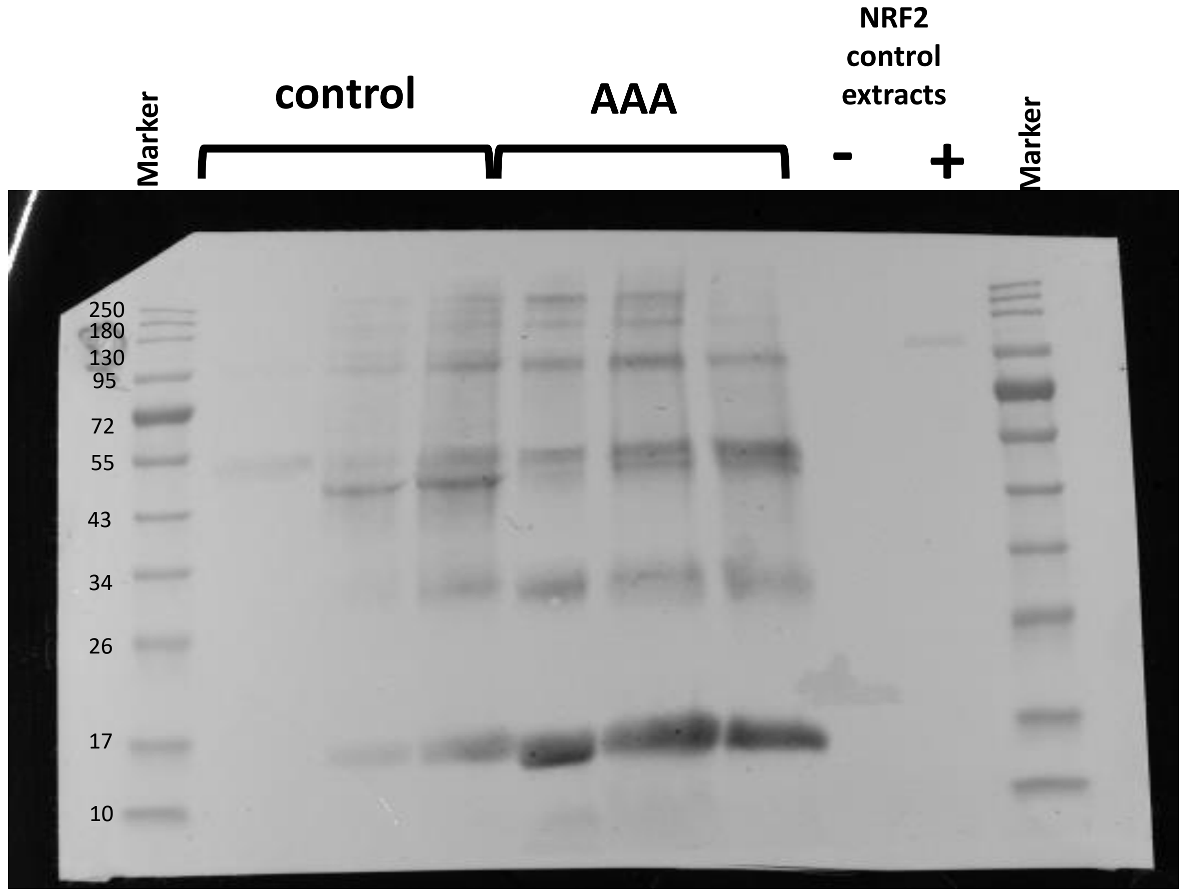

NRF2

ACTB

NRF2 ECL + ACTB ECL + Marker

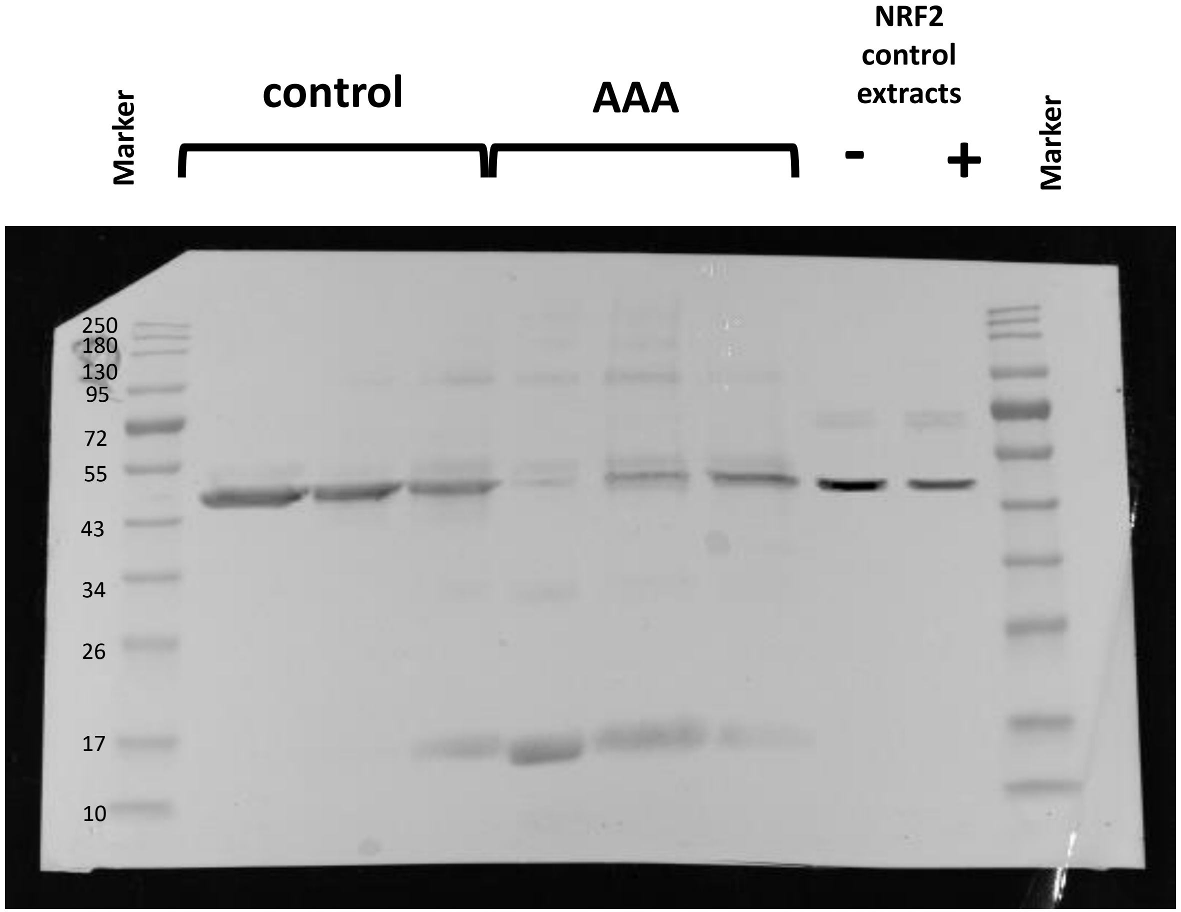

Cytoplasm

NRF2 ECL + Marker

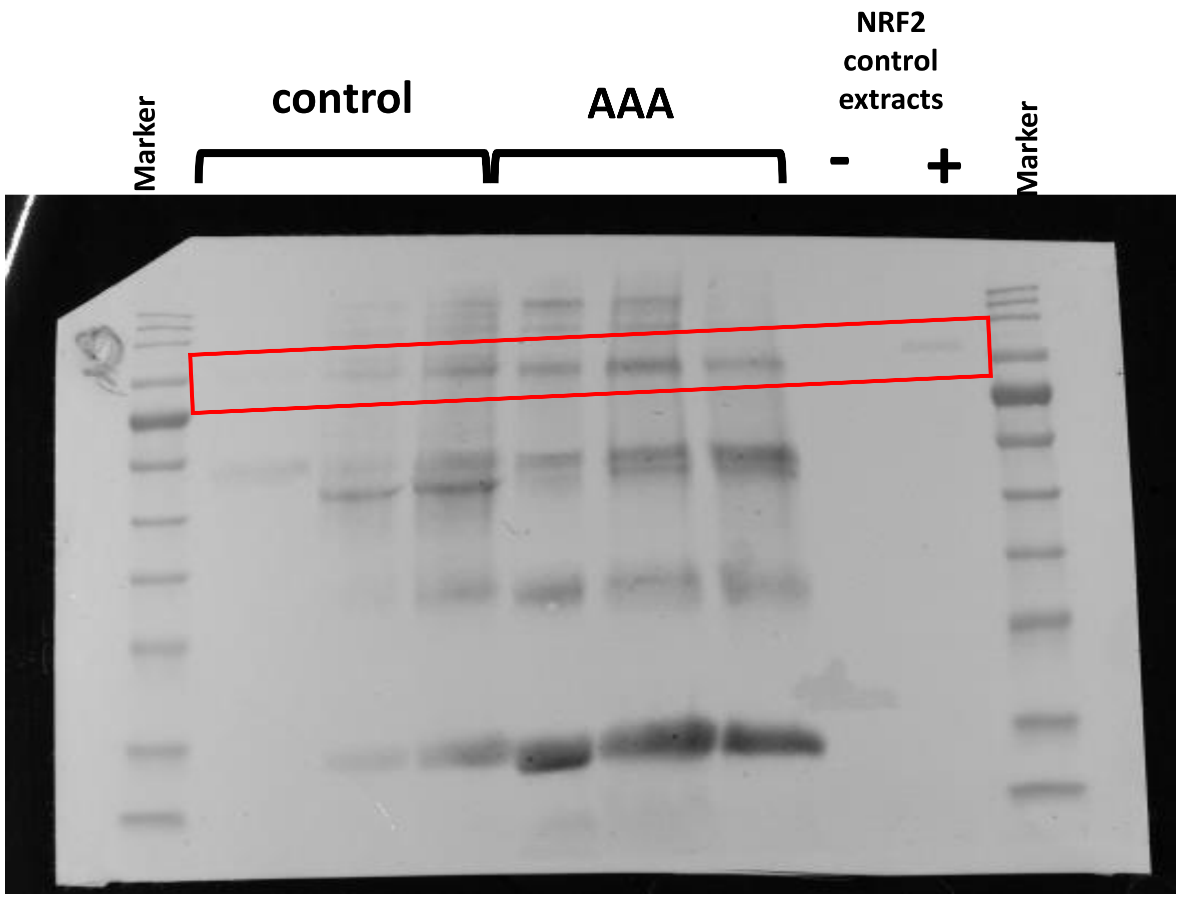

NRF2

ACTB

NRF2 ECL + ACTB ECL + Marker

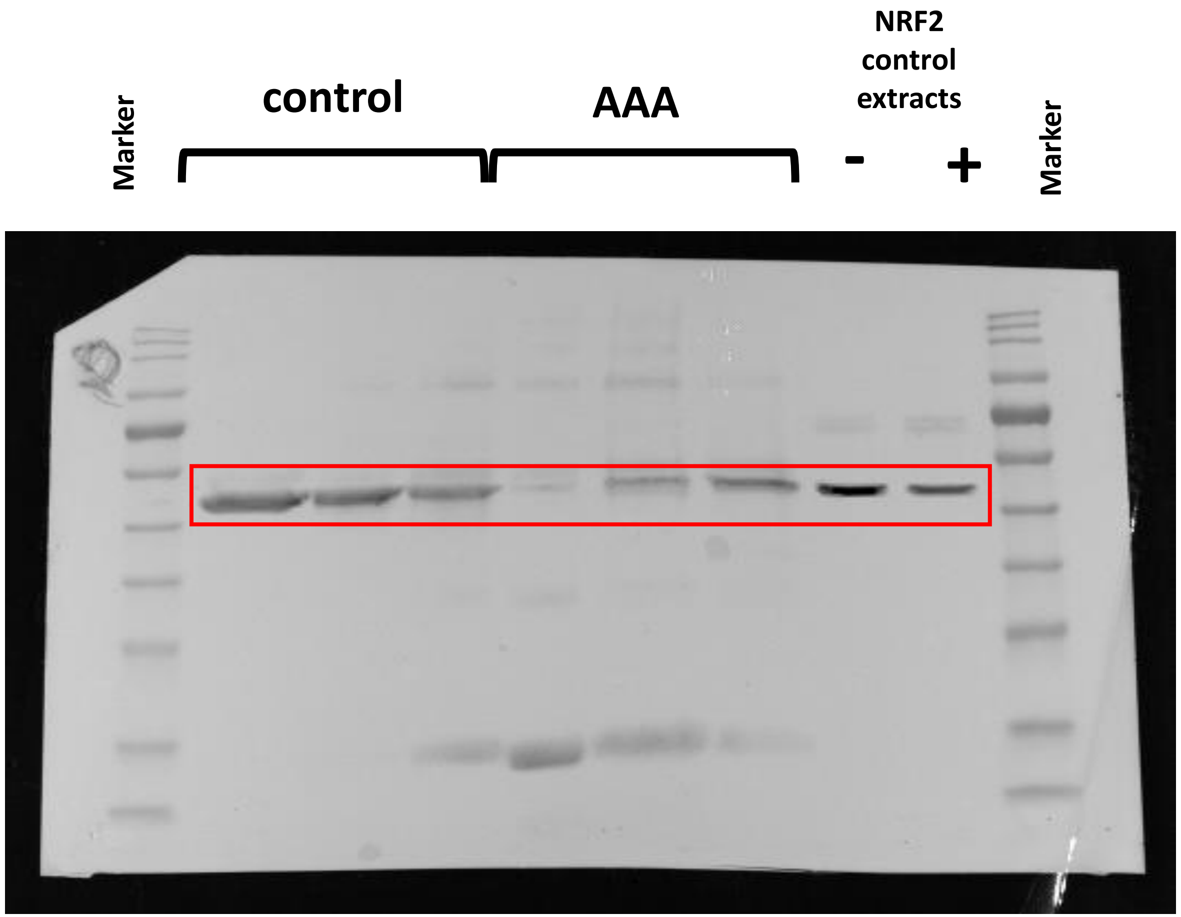

Area used for the figure

Original images of Western Blots in Supplemental Figure 3C

Please note: The images shown do not correspond to the original resolutions used for the analyses. Due to compression for display as a PDF file and the resulting reduction in pixel count, details may appear distorted.

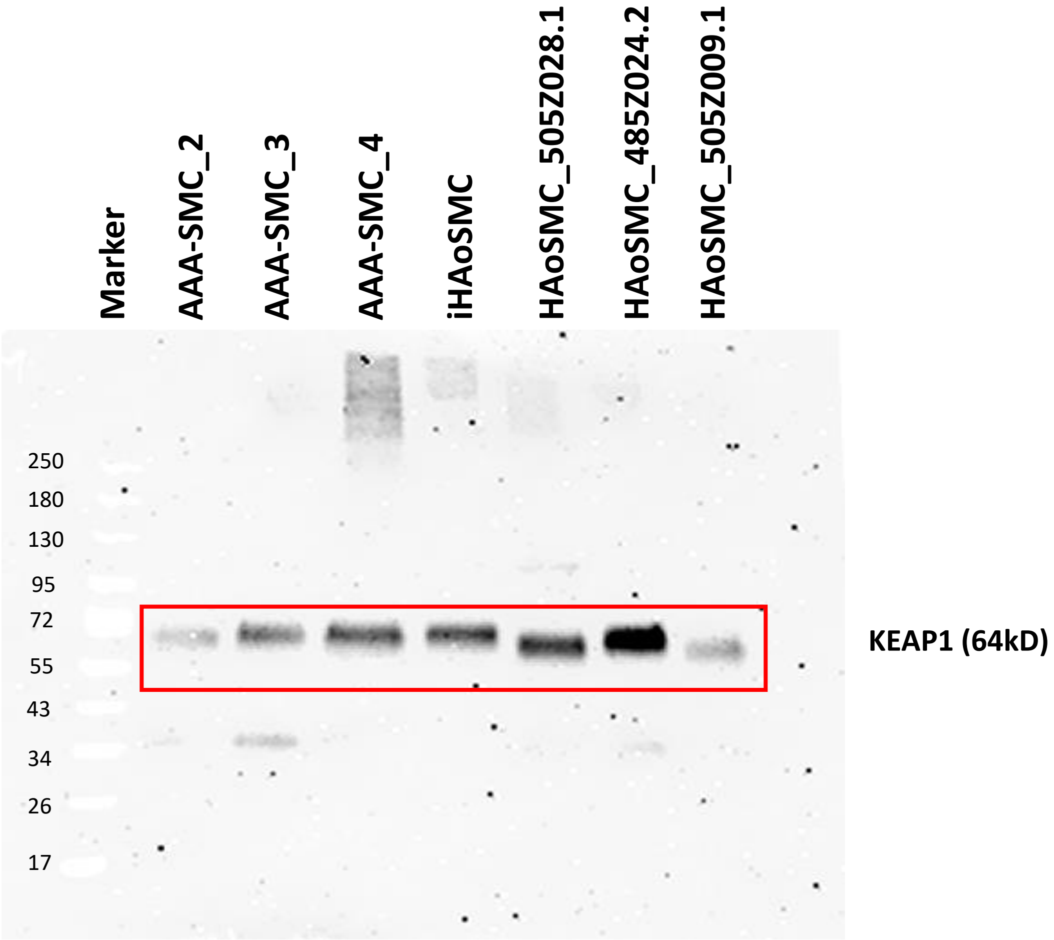
Marker
AAA-SMC_2
AAA-SMC_3
AAA-SMC_4
iHAoSMC
HAoSMC_505Z028.1
HAoSMC_485Z024.2
HAoSMC_505Z009.1
250
180
130
95
72
55
43
34
26
17
KEAP1 (64kD)

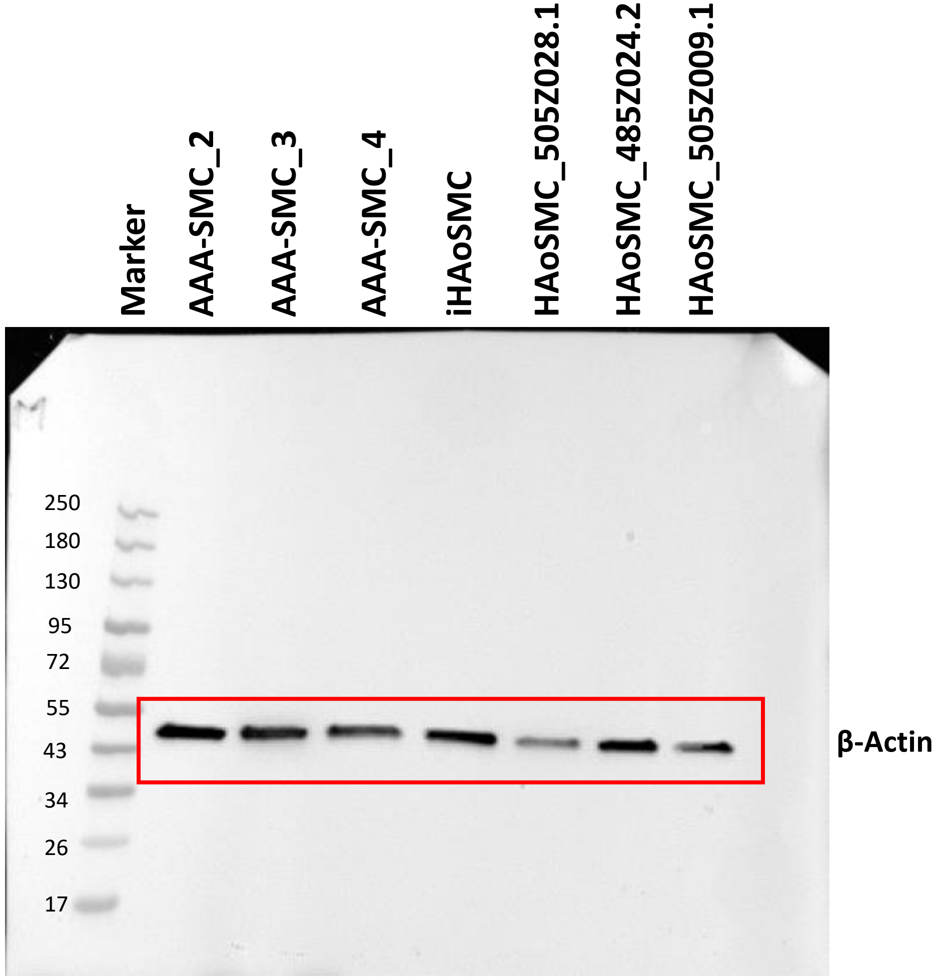
Marker
AAA-SMC_2
AAA-SMC_3
AAA-SMC_4
iHAoSMC
HAoSMC_505Z028.1
HAoSMC_485Z024.2
HAoSMC_505Z009.1
250
180
130
95
72
55
43
34
26
17
β-Actin
